# Supplementary material for: Quit attempts among tobacco users identified in the Tamil Nadu Tobacco Survey of 2015/2016: a 3 year follow-up mixed methods study
Source: BMJ Open. 2020 Sep 3;10(9):e034607. doi: 10.1136/bmjopen-2019-034607 (PMC7473619; doi:10.1136/bmjopen-2019-034607)
Supplement: Author's manuscript [file bmjopen-2019-034607.draft_revisions.pdf]

BMJ Open is committed to open peer review. As part of this commitment we make the peer review history of every article we publish publicly available.

When an article is published we post the peer reviewers' comments and the authors' responses online. We also post the versions of the paper that were used during peer review. These are the versions that the peer review comments apply to.

The versions of the paper that follow are the versions that were submitted during the peer review process. They are not the versions of record or the final published versions. They should not be cited or distributed as the published version of this manuscript.

BMJ Open is an open access journal and the full, final, typeset and author-corrected version of record of the manuscript is available on our site with no access controls, subscription charges or pay-per-view fees (http://bmjopen.bmj.com).

If you have any questions on BMJ Open's open peer review process please email info.bmjopen@bmj.com

BMJ Open

Do the tobacco users identified in the Tamil Nadu Tobacco Survey in 2015-16 continue to use tobacco? A mixed-methods study

Journal:	*BMJ Open*
Manuscript ID	bmjopen-2019-034607
Article Type:	Original research
Date Submitted by the Author:	27-Sep-2019

Complete List of Authors:	Veeraiah, Surendran; Cancer Institute-WIA, Psycho-oncology<br>Elangovan, Vidhubala; Fenivi Research Solutions<br>Tripathy, Jaya; All India Institute of Medical Sciences - Nagpur<br>Krishnamurthy, Arvind; Cancer Institute-WIA<br>Anand , T ; Indian Council of Medical Research<br>M, Mahendra; Sri Devraj Urs Medical College<br>Sudhakar, Revathy; Cancer Institute-WIA, Psycho-oncology<br>K, Niraimathi; Fenivi Research Solutions<br>Subramani, Divyarajprabhakar; Cancer Institute-WIA, Psycho-oncology<br>Rajaraman, Swaminathan; Cancer Institute-WIA<br>Elluswami, Hemanth Raj; Cancer Institute-WIA<br>Nirgude, Abhay; Yenepoya Medical College Hospital

Keywords:	PUBLIC HEALTH, EPIDEMIOLOGY, PREVENTIVE MEDICINE

SCHOLARONE™
Manuscripts

I, the Submitting Author has the right to grant and does grant on behalf of all authors of the Work (as defined in the below author licence), an exclusive licence and/or a non-exclusive licence for contributions from authors who are: i) UK Crown employees; ii) where BMJ has agreed a CC-BY licence shall apply, and/or iii) in accordance with the terms applicable for US Federal Government officers or employees acting as part of their official duties; on a worldwide, perpetual, irrevocable, royalty-free basis to BMJ Publishing Group Ltd ("BMJ") its licensees and where the relevant Journal is co-owned by BMJ to the co-owners of the Journal, to publish the Work in this journal and any other BMJ products and to exploit all rights, as set out in our licence.

The Submitting Author accepts and understands that any supply made under these terms is made by BMJ to the Submitting Author unless you are acting as an employee on behalf of your employer or a postgraduate student of an affiliated institution which is paying any applicable article publishing charge ("APC") for Open Access articles. Where the Submitting Author wishes to make the Work available on an Open Access basis (and intends to pay the relevant APC), the terms of reuse of such Open Access shall be governed by a Creative Commons licence – details of these licences and which Creative Commons licence will apply to this Work are set out in our licence referred to above.

Other than as permitted in any relevant BMJ Author's Self Archiving Policies, I confirm this Work has not been accepted for publication elsewhere, is not being considered for publication elsewhere and does not duplicate material already published. I confirm all authors consent to publication of this Work and authorise the granting of this licence.

For peer review only

Figure 1a Standard Operating Procedure for making telephone calls

The telephonic calls were placed by a trained project staff

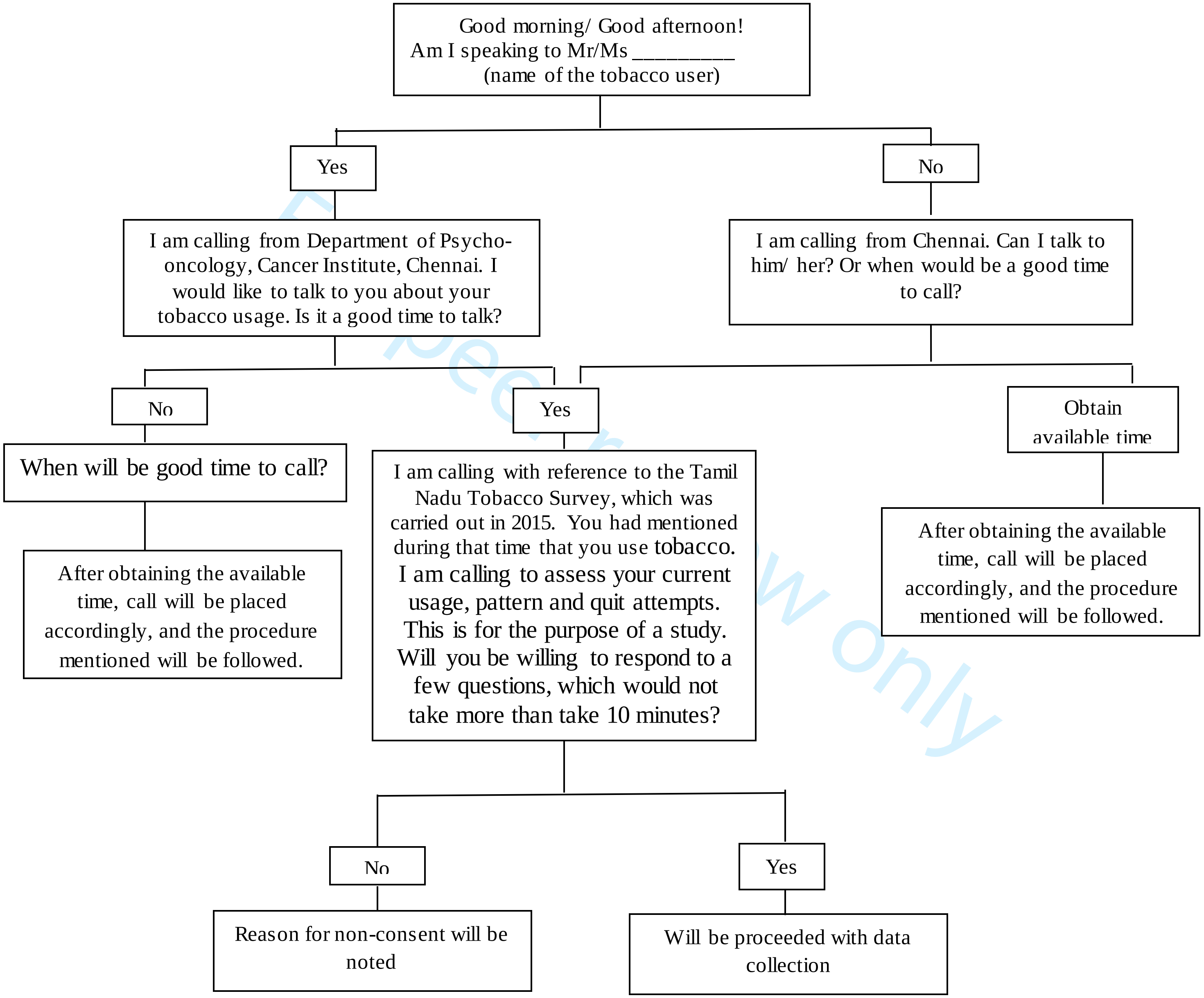

For peer review only

Figure 1b: Flow diagram depicting the status of current tobacco use and the pattern of quit attempts among tobacco users in six selected districts of Tamil Nadu previously identified in the Tamil Nadu Tobacco Survey (TNTS) (2015-16)

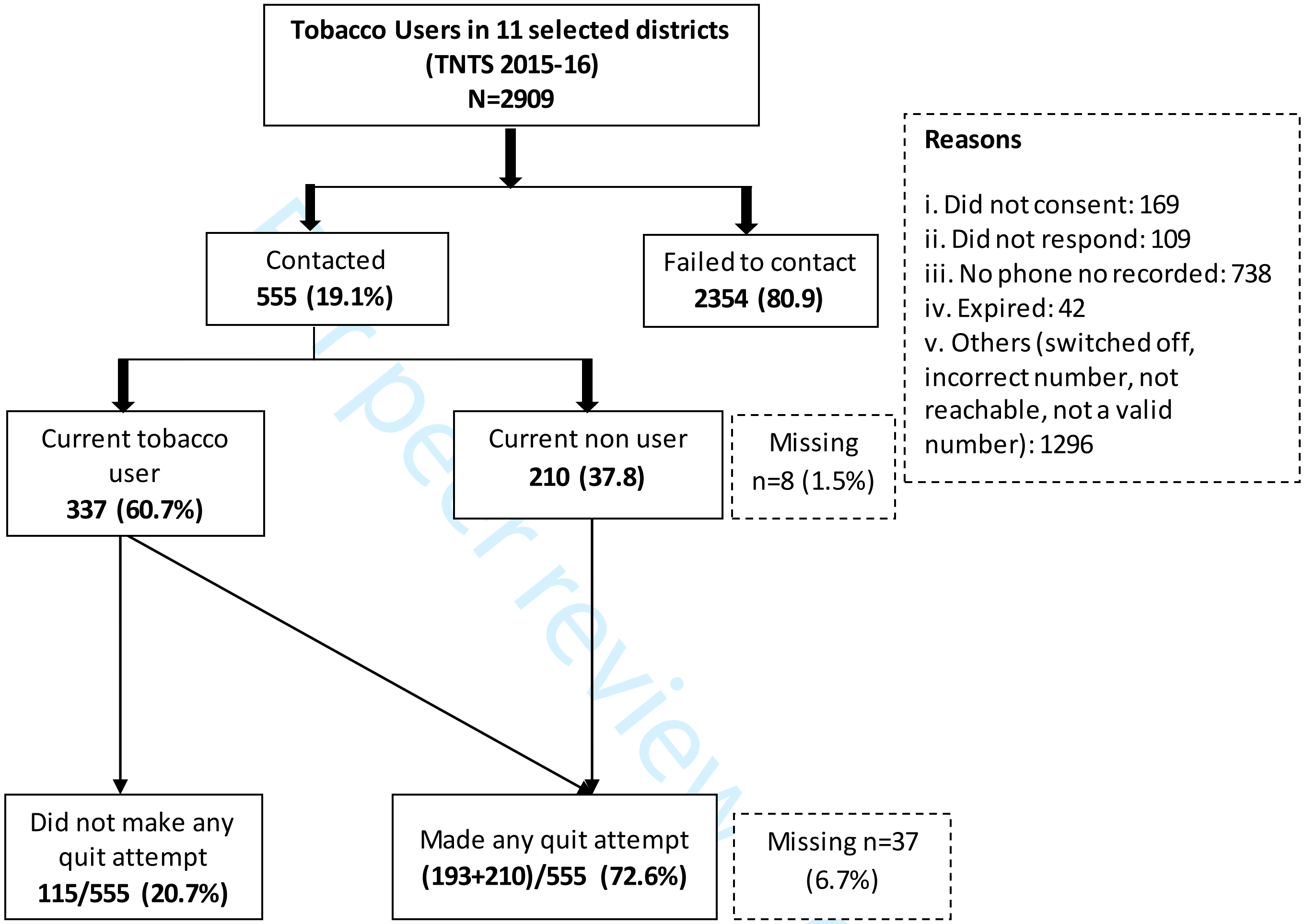

For peer review only

Figure 2 Enablersof quitting tobacco and sustaining it among the tobacco users in three selected districts of Tamil Nadu, 2019

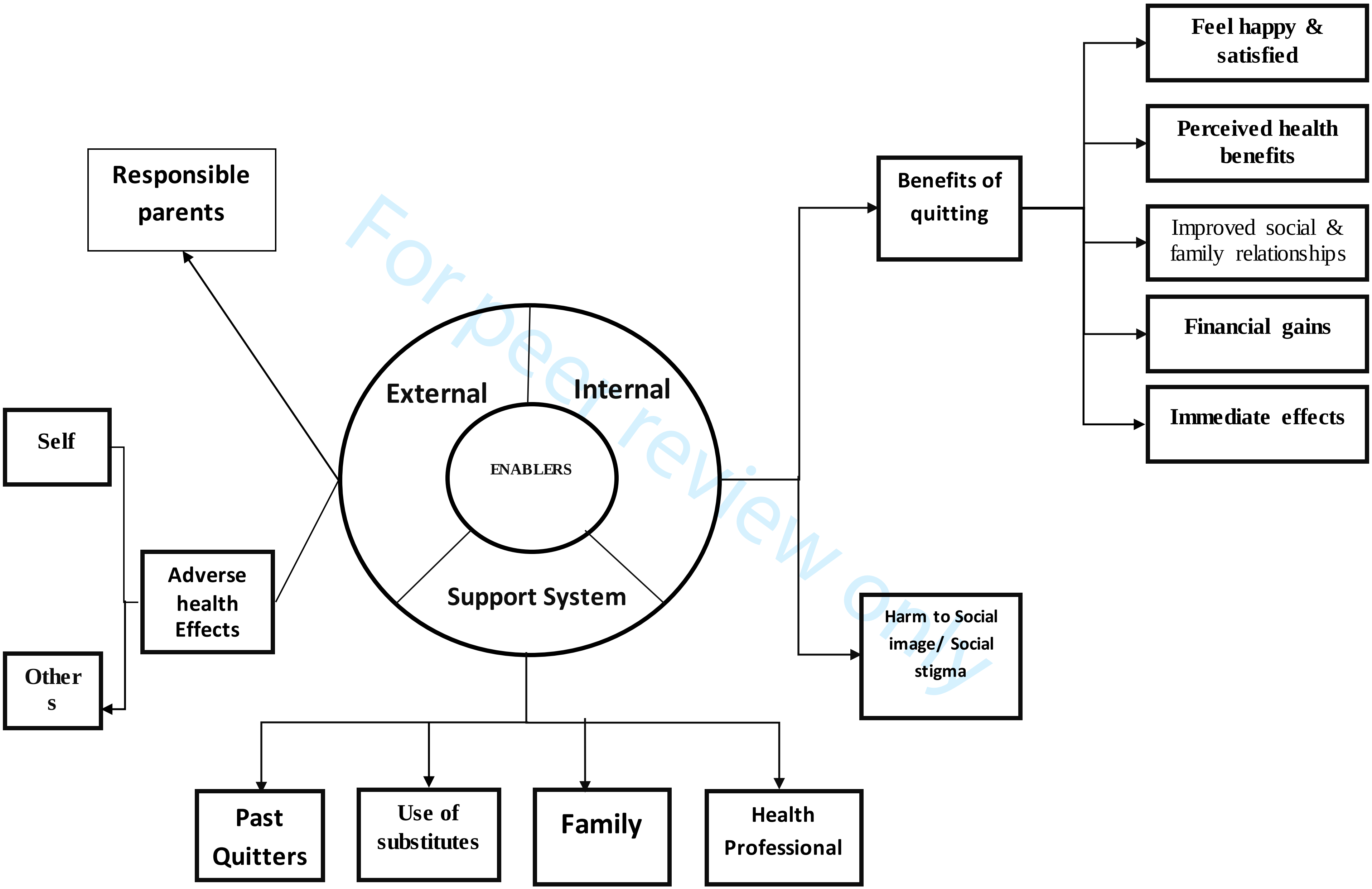

Figure 3 Barriers of quitting tobacco and sustaining it among the tobacco users in three selected districts of Tamil Nadu, 2019

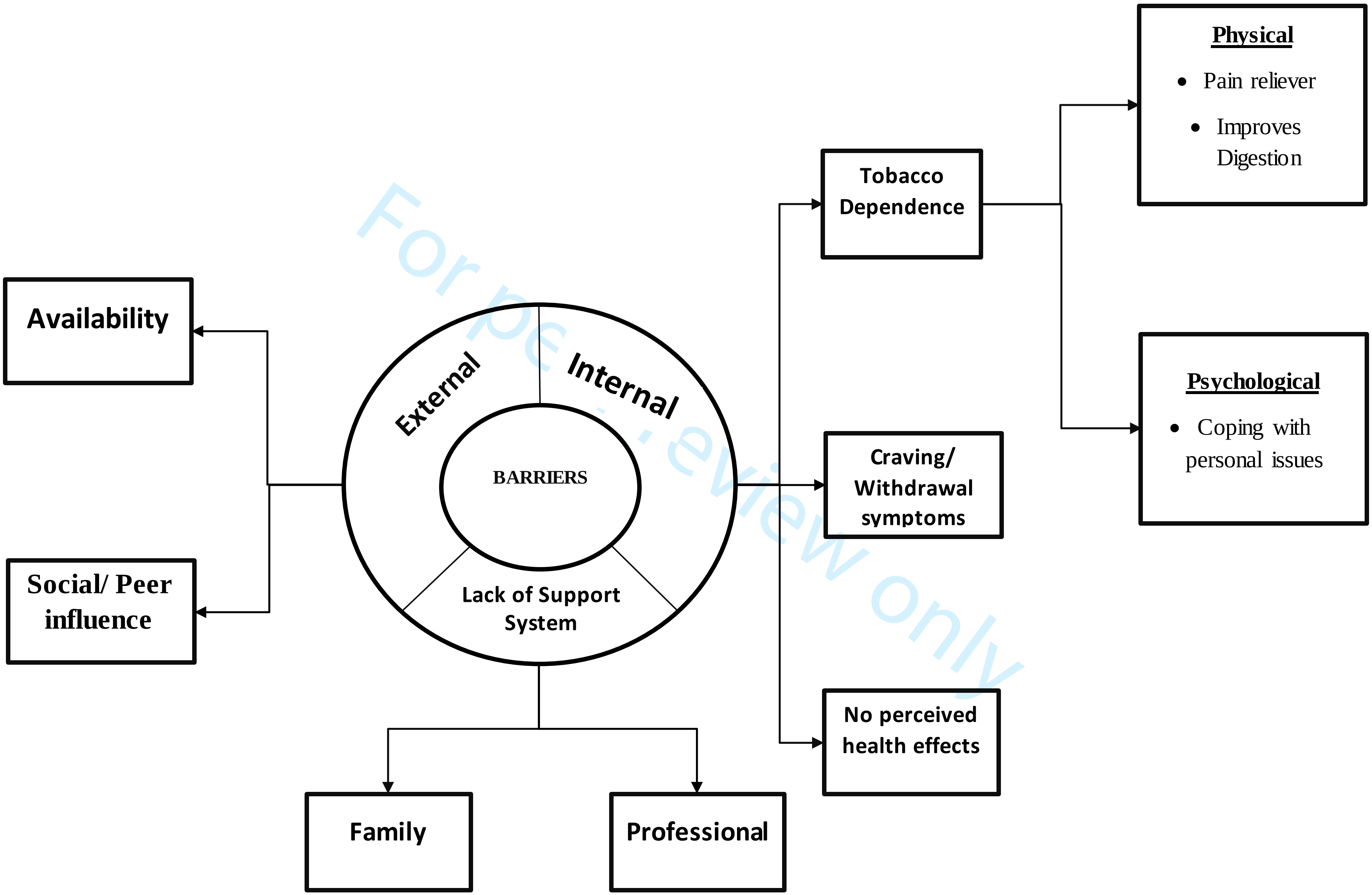

STROBE Statement—Checklist of items that should be included in reports of ***cohort studies***

	Item No	Recommendation	Page No
**Title and abstract**	1	(*a*) Indicate the study's design with a commonly used term in the title or the abstract	1
		(*b*) Provide in the abstract an informative and balanced summary of what was done and what was found	3
**Introduction**			
Background/rationale	2	Explain the scientific background and rationale for the investigation being reported	4
Objectives	3	State specific objectives, including any prespecified hypotheses	5
**Methods**			
Study design	4	Present key elements of study design early in the paper	6
Setting	5	Describe the setting, locations, and relevant dates, including periods of recruitment, exposure, follow-up, and data collection	6
Participants	6	(*a*) Give the eligibility criteria, and the sources and methods of selection of participants. Describe methods of follow-up	6
		(*b*) For matched studies, give matching criteria and number of exposed and unexposed	
Variables	7	Clearly define all outcomes, exposures, predictors, potential confounders, and effect modifiers. Give diagnostic criteria, if applicable	7
Data sources/ measurement	8*	For each variable of interest, give sources of data and details of methods of assessment (measurement). Describe comparability of assessment methods if there is more than one group	7
Bias	9	Describe any efforts to address potential sources of bias	
Study size	10	Explain how the study size was arrived at	7
Quantitative variables	11	Explain how quantitative variables were handled in the analyses. If applicable, describe which groupings were chosen and why	7
Statistical methods	12	(*a*) Describe all statistical methods, including those used to control for confounding	9
		(*b*) Describe any methods used to examine subgroups and interactions	
		(*c*) Explain how missing data were addressed	
		(*d*) If applicable, explain how loss to follow-up was addressed	
		(*e*) Describe any sensitivity analyses	
**Results**			
Participants	13*	(a) Report numbers of individuals at each stage of study—eg numbers potentially eligible, examined for eligibility, confirmed eligible, included in the study, completing follow-up, and analysed	10
		(b) Give reasons for non-participation at each stage	10
		(c) Consider use of a flow diagram	10
Descriptive data	14*	(a) Give characteristics of study participants (eg demographic, clinical, social) and information on exposures and potential confounders	10
		(b) Indicate number of participants with missing data for each variable of interest	10
		(c) Summarise follow-up time (eg, average and total amount)	10
Outcome data	15*	Report numbers of outcome events or summary measures over time	10

For peer review only

For peer review only - http://bmjopen.bmj.com/site/about/guidelines.xhtml

Main results	16	(*a*) Give unadjusted estimates and, if applicable, confounder-adjusted estimates and their precision (eg, 95% confidence interval). Make clear which confounders were adjusted for and why they were included<br>(*b*) Report category boundaries when continuous variables were categorized<br>(*c*) If relevant, consider translating estimates of relative risk into absolute risk for a meaningful time period	11
Other analyses	17	Report other analyses done—eg analyses of subgroups and interactions, and sensitivity analyses	
**Discussion**			
Key results	18	Summarise key results with reference to study objectives	16
Limitations	19	Discuss limitations of the study, taking into account sources of potential bias or imprecision. Discuss both direction and magnitude of any potential bias	16
Interpretation	20	Give a cautious overall interpretation of results considering objectives, limitations, multiplicity of analyses, results from similar studies, and other relevant evidence	16
Generalisability	21	Discuss the generalisability (external validity) of the study results	16
**Other information**			
Funding	22	Give the source of funding and the role of the funders for the present study and, if applicable, for the original study on which the present article is based	17

For peer review only

For peer review only - http://bmjopen.bmj.com/site/about/guidelines.xhtml

COREQ (COnsolidated criteria for REporting Qualitative research) Checklist

A checklist of items that should be included in reports of qualitative research. You must report the page number in your manuscript where you consider each of the items listed in this checklist. If you have not included this information, either revise your manuscript accordingly before submitting or note N/A.

Topic	Item No.	Guide Questions/Description	Reported on Page No.
**Domain 1: Research team and reflexivity**			
Personal characteristics			
Interviewer/facilitator	1	Which author/s conducted the interview or focus group?	8
Credentials	2	What were the researcher's credentials? E.g. PhD, MD	M.Phil., PhD & M.Phil
Occupation	3	What was their occupation at the time of the study?	1
Gender	4	Was the researcher male or female?	
Experience and training	5	What experience or training did the researcher have?	8
Relationship with participants			
Relationship established	6	Was a relationship established prior to study commencement?	6
Participant knowledge of the interviewer	7	What did the participants know about the researcher? e.g. personal goals, reasons for doing the research	8
Interviewer characteristics	8	What characteristics were reported about the inter viewer/facilitator? e.g. Bias, assumptions, reasons and interests in the research topic	8
**Domain 2: Study design**			
Theoretical framework			
Methodological orientation and Theory	9	What methodological orientation was stated to underpin the study? e.g. grounded theory, discourse analysis, ethnography, phenomenology, content analysis	9
Participant selection			
Sampling	10	How were participants selected? e.g. purposive, convenience, consecutive, snowball	6
Method of approach	11	How were participants approached? e.g. face-to-face, telephone, mail, email	8
Sample size	12	How many participants were in the study?	8
Non-participation	13	How many people refused to participate or dropped out? Reasons?	10
Setting			
Setting of data collection	14	Where was the data collected? e.g. home, clinic, workplace	8
Presence of non-participants	15	Was anyone else present besides the participants and researchers?	8
Description of sample	16	What are the important characteristics of the sample? e.g. demographic data, date	10
Data collection			
Interview guide	17	Were questions, prompts, guides provided by the authors? Was it pilot tested?	8
Repeat interviews	18	Were repeat inter views carried out? If yes, how many?	
Audio/visual recording	19	Did the research use audio or visual recording to collect the data?	8
Field notes	20	Were field notes made during and/or after the inter view or focus group?	8
Duration	21	What was the duration of the inter views or focus group?	8

For peer review only

For peer review only - http://bmjopen.bmj.com/site/about/guidelines.xhtml

Data saturation	22	Was data saturation discussed?	8
Transcripts returned	23	Were transcripts returned to participants for comment and/or	8

Topic	Item No.	Guide Questions/Description	Reported on Page No.
		correction?	
**Domain 3: analysis and findings**			
Data analysis			
Number of data coders	24	How many data coders coded the data?	9
Description of the coding tree	25	Did authors provide a description of the coding tree?	11
Derivation of themes	26	Were themes identified in advance or derived from the data?	9
Software	27	What software, if applicable, was used to manage the data?	9
Participant checking	28	Did participants provide feedback on the findings?	8
Reporting			
Quotations presented	29	Were participant quotations presented to illustrate the themes/findings? Was each quotation identified? e.g. participant number	11
Data and findings consistent	30	Was there consistency between the data presented and the findings?	15
Clarity of major themes	31	Were major themes clearly presented in the findings?	11
Clarity of minor themes	32	Is there a description of diverse cases or discussion of minor themes?	11

For peer review only

Developed from: Tong A, Sainsbury P, Craig J. Consolidated criteria for reporting qualitative research (COREQ): a 32-item checklist for interviews and focus groups. *International Journal for Quality in Health Care*. 2007. Volume 19, Number 6: pp. 349 – 357

Once you have completed this checklist, please save a copy and upload it as part of your submission. DO NOT include this checklist as part of the main manuscript document. It must be uploaded as a separate file.

For peer review only

BMJ Open

Quit attempts amongst tobacco users identified in the Tamil Nadu Tobacco Survey of 2015-16: A 3 year follow-up mixed methods study

Journal:	*BMJ Open*
Manuscript ID	bmjopen-2019-034607.R1
Article Type:	Original research
Date Submitted by the Author:	20-Apr-2020
Complete List of Authors:	Veeraiah, Surendran; Cancer Institute-WIA, Psycho-oncology<br>Elangovan, Vidhubala; Fenivi Research Solutions<br>Tripathy, Jaya; All India Institute of Medical Sciences - Nagpur<br>Krishnamurthy, Arvind; Cancer Institute-WIA<br>Anand , T ; Indian Council of Medical Research<br>M, Mahendra; Sri Devraj Urs Medical College<br>Sudhakar, Revathy; Cancer Institute-WIA, Psycho-oncology<br>K, Niraimathi; Fenivi Research Solutions<br>Subramani, Divyarajprabhakar; Cancer Institute-WIA, Psycho-oncology<br>Rajaraman, Swaminathan; Cancer Institute-WIA<br>Elluswami, Hemanth Raj; Cancer Institute-WIA<br>Nirgude, Abhay; Yenepoya Medical College Hospital
**Primary Subject Heading**:	Public health
Secondary Subject Heading:	Smoking and tobacco, Epidemiology
Keywords:	PUBLIC HEALTH, EPIDEMIOLOGY, PREVENTIVE MEDICINE

SCHOLARONE™
Manuscripts

I, the Submitting Author has the right to grant and does grant on behalf of all authors of the Work (as defined in the below author licence), an exclusive licence and/or a non-exclusive licence for contributions from authors who are: i) UK Crown employees; ii) where BMJ has agreed a CC-BY licence shall apply, and/or iii) in accordance with the terms applicable for US Federal Government officers or employees acting as part of their official duties; on a worldwide, perpetual, irrevocable, royalty-free basis to BMJ Publishing Group Ltd ("BMJ") its licensees and where the relevant Journal is co-owned by BMJ to the co-owners of the Journal, to publish the Work in this journal and any other BMJ products and to exploit all rights, as set out in our licence.

The Submitting Author accepts and understands that any supply made under these terms is made by BMJ to the Submitting Author unless you are acting as an employee on behalf of your employer or a postgraduate student of an affiliated institution which is paying any applicable article publishing charge ("APC") for Open Access articles. Where the Submitting Author wishes to make the Work available on an Open Access basis (and intends to pay the relevant APC), the terms of reuse of such Open Access shall be governed by a Creative Commons licence – details of these licences and which Creative Commons licence will apply to this Work are set out in our licence referred to above.

Other than as permitted in any relevant BMJ Author's Self Archiving Policies, I confirm this Work has not been accepted for publication elsewhere, is not being considered for publication elsewhere and does not duplicate material already published. I confirm all authors consent to publication of this Work and authorise the granting of this licence.

For peer review only

Title Page

Quit attempts amongst tobacco users identified in the Tamil Nadu Tobacco Survey of 2015-16: A 3 year follow-up mixed methods study

Short running title: Successful tobacco quit attempts: enablers and barriers

Authors and affiliations

Surendran Veeraiah[1], E Grace Sahaya Vidhubala[2], Jaya Prasad Tripathy[3], Arvind Krishnamurthy[1], Tanu Anand[4], Mahendra M[5], Revathy Sudhakar[1], Niraimathi K[2], Divyaraj Prabhakar[1], Swaminathan R[1], E Hemanth Raj[1], Abhay Nirgude[6]

1 Cancer Institute (WIA), Chennai, India

2 Fenivi Research Solutions, Chennai, India

3 All India Institute of Medical Sciences, Nagpur, India

4 Indian Council of Medical Research, Department of Health Research, Ministry of Health and Family Welfare, New Delhi

5 Sri Devraj Urs Medical College, Kolar, Karnataka

6 Yenepoya Medical College, Mangalore, India

Details of the corresponding author

Surendran Veeraiah

Associate Professor and Head

Department of Psycho-oncology and Resource Centre for Tobacco Control

Cancer Institute (WIA),

38, Sardar Patel Road, Adyar, Chennai,

Tamil Nadu, India. PIN 600036

suren.psy@gmail.com

v.surendran@cancerinstitutewia.org

For peer review only

Word Count: Manuscript: 3941; Abstract: 278

Number of tables: 5

Number of figures: 3

Number of references: 26

Keywords- Quit attempt, Tobacco use, Enablers & Barriers, SORT-IT

For peer review only

Abstract

Objectives

To determine current tobacco use in 2018-19, quit attempts made and explore the enablers and barriers in quitting tobacco among tobacco users identified in the Tamil Nadu Tobacco Survey (TNTS) in 2015-16.

Setting

TNTS was conducted in 2015-16 throughout the state of TN in India covering 111363 individuals. Tobacco prevalence was found to be xx% (n=)

Participants

All tobacco users in eleven districts of TN identified by TNTS (n=2909) were tracked after three years by telephone. In-depth interviews were conducted in a sub-sample to understand the enablers and barriers in quitting.

Primary and Secondary Outcomes

Current tobacco use status, any quit attempt and successful quit rate were the primary outcomes, while barriers and enablers in quitting were considered as secondary outcomes.

Results

Among the 2909 tobacco users identified in TNTS 2015-16, only 724 (24.9%) could be contacted by telephone, of which 555 (76.7%) consented. Of those who consented, 210 (37.8%) were currently not using tobacco (i.e. successfully quit) and 337 (60.7%) continued to use any form of tobacco. Of current tobacco users, 115 (34.1%) never made any quit attempt and 193 (57.3.8%) have made any attempt to quit. Those using smoking form of tobacco products (aRR=1.2, 95% CI: 1.1-1.4) and exposure to smoke at home (aRR=1.2, 95% CI: 1.1-1.3) were found to be positively associated with continued tobacco use (failed or no quit attempt). Support from family and perceived health benefits are key enablers, while peer influence, high dependence and lack of professional help are some of the barriers to quitting.

Conclusion

Two-thirds of the tobacco users continue to use tobacco in the last 3 years. While tobacco users are well aware of the ill-effects of tobacco, various intrinsic and extrinsic factors play a major role as a facilitator and lack of the same act as a barrier to quit.

Strengths and limitations of this study

- This is the first such study to attempt a follow-up of tobacco users identified in previous survey to understand their current tobacco use status and quit attempts.
- The study involved telephone survey to contact the tobacco users
- The mixed-methods design enabled estimation of quit rates and understanding the enablers and barriers in quitting tobacco.
- A major limitation of this study was the poor response rate of the telephonic survey which might have introduced responder bias.
- There was no objective means of verifying the responses received by telephone.

INTRODUCTION

The tobacco epidemic continues to be a major public health concern with nearly 1.4 billion tobacco users worldwide. It is one of the most important preventable causes of premature death in the world claiming more than 8 million lives each year.(1,2)

To address the growing tobacco menace, the World Health Organization Framework Convention on Tobacco Control (WHO FCTC) came into force in 2005. This international treaty has been ratified by 181 countries, and provides a roadmap for the countries to adopt and implement tobacco control measures. Article 14 of WHO FCTC mentions the dissemination of comprehensive guidelines based on scientific evidence to promote tobacco cessation. To assist in country-level implementation of the WHO FCTC, WHO also introduced a package of six technical measures termed as the MPOWER strategy, where 'O' stands for 'offer help to quit tobacco use' which is one of the key components of this strategy.

It is beyond any doubt that quitting tobacco is one of the most effective ways of saving lives and improving overall well-being. Majority of the smokers regret ever starting to smoke and want to quit.(3) However, quitting smoking remains difficult primarily because of the

For peer review only

addictiveness of nicotine in tobacco, along with other social and contextual factors.(4–6)It is reported that only about 3-5% of unassisted quit attempts are successful.(7,8)

In India, the prevalence of tobacco use in any form is 29% of all adults (42% of men and 14% of women).(9) Tobacco use contributes to nearly 10% of all deaths in the country with more than 1 million deaths in 2016.(10) According to the Global Adult Tobacco Survey 2016-17 (GATS) in India, more than half of current tobacco users were planning or thinking of quitting tobacco use.(9) However, we do not know how many of them actually made a quit attempt or went on to become a successful quitter. Several other large nationally representative cross-sectional studies such as GATS, National Health Family Surveys etc. have examined tobacco prevalence. However, these surveys are cross-sectional in nature with limited cohort-wise assessment of tobacco users and their quitting behaviour over a period of time.

A cross-sectional household tobacco survey, Tamil Nadu Tobacco Survey (TNTS), was conducted in 2015-16 in the state of Tamil Nadu, South India by the Cancer Institute (Women's India Association), Chennai, India to provide reliable state and district-wise estimates of tobacco use.(11) The survey covered nearly 100 000adults (>15 years) in all 32 districts across the state. The results of the survey showed that 5.2% were current tobacco users and about one in every five tobacco users reported to have intention to quit tobacco use in the next one month. But how many of them actually quit and how many of those who made a quit attempt were successful, is unknown.

In order to answer these questions, we did a follow-up of those who were identified as tobacco users in the TNTS three years post-survey by telephone to understand their current tobacco use status and any quit attempts made in the last three years. After quantitatively assessing tobacco use status, quit rates and quit attempts among previous tobacco users, it is also useful to understand the enablers that motivated and barriers they faced in quitting or attempting to quit tobacco through a qualitative approach. This will help design a tailored package of cessation and counselling intervention. Hence, a sequential explanatory mixed-method design was adopted for this study wherein the sample for qualitative study was a subset of the quantitative sample.

The specific objectives of the study were:

For peer review only

1. Among the tobacco users previously identified in the TNTS in 2015-16, determine the number and proportion who could be contacted through a telephone survey in 2018-19 and compare their characteristics with those who could not be contacted

2. Among those contacted by telephone in 2018-19, determine the number and proportion who i) continue to use tobacco (smoking and/or smokeless) i.e. failed or no quit attempt, ii) made a successful quit attempt, iii) made any quit attempt,

3. Explore the barriers and enablers in making and sustaining a quit attempt.

METHODS

Study Design

This study employed a sequential explanatory mixed-methods design with a cohort study design as the quantitative component followed by a descriptive qualitative component. (12) The quantitative cohort study was a follow-up of assessment of tobacco users identified during the TNTS in 2015-16 to assess their current tobacco use and quit attempts.

Setting

General setting:

In order to tackle the burden of tobacco use in the country, the Ministry of Health and Family Welfare launched a network of 19 tobacco cessation clinics (TCCs) in India in 2002 with the support from WHO. These clinics offer a wide variety of behavioural (brief advice, 5A's and 5R's, individual/group counselling) and pharmacological interventions (Nicotine Replacement Therapy: nicotine patch, gum, inhaler, spray and non-nicotine replacement therapy: bupropion, varenicline) for tobacco cessation free of cost. A combination of behavioural and pharmacotherapy is generally considered the best approach for treating tobacco dependence. Subsequently, the National Tobacco Control Programme was launched in 2007-08 to be implemented by Tobacco Control Cells at the national, state and district level. Under this program, there is also a provision of setting up Tobacco Cessation Services at the district level. India has also launched quitline (toll free helpline service) and Cessation program wherein tobacco users can register to receive tailored cessation advice via mobile messages.

Tamil Nadu (TN)is the sixth largest state by population with about 72 million people.(13) It has 32 administrative districts. With nearly half of the population residing in urban areas, it has a high literacy rate of 80% (14).In TN, according to GATS 2, nearly 20% use tobacco in any

For peer review only

form, of whom 9.5% are smokers, 9.5% are smokeless tobacco users and remaining 1% use both.(9)

Specific setting:

Tamil Nadu Tobacco Survey (TNTS) 2015-16

The TNTS identified 111,363 eligible individuals aged 15 years and above, from 32,945 households across all 32 districts in TN. Of these, 99,825 individuals contacted door to door, responded, with the response rate being 89.2%. All these individuals were assessed for tobacco use, exposure to second hand smoke, quit behaviour, impact of pictorial warnings and other tobacco control legislations.

Survey sampling methodology

Under TNTS, each of the 32 districts was divided into urban and rural areas, whereas Chennai city was divided into 15 zones, each zone further sub-divided into slum and non-slum. The estimated sample was divided among all urban and rural areas of districts, slums and non-slum areas of zones in Chennai city using Probability Proportional to Size sampling [6]. Data were collected during 2015-2016. The details of the survey methodology are given elsewhere.(11,15,16)

Study population/Sampling frame

The study population for both the quantitative and qualitative component included all the identified tobacco users (n=5208) from the TNTS 2015-16. The quantitative sample was recruited by a telephone survey. The qualitative sample is a subset of the quantitative sample.

Sample size

Assuming that about 6% of tobacco users make a successful quit attempt, with 2% absolute precision and 80% power, sample size was calculated to be 610. Assuming 33% response rate from our previous experience (this being a telephonic survey), the final sample size was estimated to be 2025. These participants were recruited from the original TNTS survey conducted in 2015-16 by telephonic survey.

Data variables, sources of data and data collection

Quantitative

For peer review only

Data were collected from two sources: a) TNTS database (already collected in 2015-16) and b) Telephonic survey (conducted in 2018-19). A structured questionnaire was used to collect information by telephone survey with the respondents of the original TNTS survey, with the items broadly covering areas such as current tobacco usage (both smoking and/or smokeless), quit attempt(s) and their duration and their intention to quit. In addition, socio-demographic and tobacco use related variables were extracted from the TNTS. Reported tobacco users of TNTS (N=2909) in 11 districts of TN were contacted through telephone by a team of trained project staff at the Cancer Institute, Chennai. A Standard Operating Procedure (SOP) was prepared and followed for telephone survey (**Figure 1a**). Briefly, at every instance, each tobacco user was contacted a maximum of three times at an interval of 30 minutes in a day. After two calls, a standardized text message was sent stating the details of the caller and the purpose of the call. Subsequently, the tobacco user was called with an interval of 30 minutes after the text message. This process was repeated again after 7 days (if no contact made in the previous attempt) before labeling it as an unsuccessful contact. In addition to the name and ID of the patient, response to each call was recorded by the project staff using a separate sheet as: no response, disconnected the call, number not reachable, number invalid, refused to share information, busy schedule, responded to the call and so on. Respondents who were contacted and consented to participate were briefed about the purpose of the call. On obtaining verbal consent, the questions were administered over telephone and the responses were recorded on a structured questionnaire. The telephone calls were not recorded as it might affect the responses of the participant. The participants might be reluctant to share their experiences, if the calls are recorded, also referred to as Hawthorne effect. However, the telephone survey was monitored by an individual not associated with the current research for interviewer compliance with the protocol described above. Verbal feedback was given continuously to improve and finetune the process.

Qualitative

The Principal Investigator (PI) (Ph.D. in Psychology) and the co-PI (M.Phil.) who are trained in qualitative research methods conducted In-Depth Interviews (IDIs)after obtaining consent of the participants.The IDIs were conducted in regional language (*Tamil*) by telephone using an interview guide with open ended questions related to the quit attempts made, method of quitting and motivation to quit and barriers/motivators for failed or successful quit attempts. This data was collected separately and not as a part of the quantitative data collection with all the participants, due to time constraint. The interviews were audio recorded (after obtaining

consent) and verbatim notes were also taken during the interview. Each interview lasted for around 30 minutes. After the interview was over, the summary of the interviews was read back to the participants to ensure participant validation. Since it was a telephone interview, no incentives were provided for the participants. A total of 8-10 IDIs were planned to be conducted in each district to cover those who made a successful quit attempt, failed attempt and did not made a quit attempt. It was planned to cover both smokers and smokeless tobacco users in the sample.

Operational Definitions (11)

Quit attempt

Any attempt at tobacco cessation that lasts for 1 or more than one day, including both self-attempt as well as attempt with professional help.

Current tobacco users

Tobacco users, who reported using any form of tobacco daily or occasionally for more than one month prior to the interview.

Sampling

Quantitative

All tobacco users identified in TNTS 2015-16 in 11 purposively selected districts namely Chennai, Coimbatore, Kanchipuram, Madurai, Tirunelveli and Tiruvallur, Viluppuram, Pudukkottai, Kanyakumari, Tiruppur and Erode were recruited (n=2909) consecutively. These districts were purposively selected to ensure wider geographical coverage.

Qualitative

The sample for IDIs included a conveniently selected lot of tobacco users identified through TNTS 2015-16, residing in Chennai, Kanchipuram and Thiruvallur Districts. The participants of the telephone survey were divided into three groups: i) those who made a failed quit attempt (n=10), ii) made a successful quit attempt (n=10), and iii) those who did not make any attempt (n=6). Around 6-10 IDIs were conducted in each of the three districts (n=26) from three groups. Maximum variation sampling was used to include both smokers and smokeless tobacco users from different age groups. Data saturation was practiced using informational redundancy approach. Further interviews were discontinued if no new information was obtained pertaining to the major themes. However, there was inadequate response from the third group where the participants did not make any quit attempt.

For peer review only

Analysis and Statistics

Quantitative

Quantitative data were double entered and validated using EpiData entry (version 3.0) and analysed using EpiData analysis (version 2.2.2.183, EpiData Association, Odense, Denmark) and STATA version 13.0. The key outcome indicators were current tobacco use and quit attempt. Chi-square test was used to find the association between various socio-demographic, tobacco use related variables with the current tobacco use. Binomial regression was used to explore the factors associated with tobacco use. Adjusted Relative Risks (aRRs) with 95% confidence intervals was used to measure the strength of the association.

Qualitative

The audio recorded interviews were transcribed manually in local language, Tamil, by the PI (SV) and the co-PI (RS) as soon the interviews were over. The transcripts were read multiple times by two investigators (SV and RS) before coding. Thematic analysis following the six-phase approach by Braun & Clarke (2006) was undertaken to analyse the transcripts.(17) A hierarchical codebook was developed by two study investigators (SV and RS) by synthesizing codes emerging directly from the transcripts (inductive) and from the topic guides (deductive).The initial coding was done independently by the investigators after going through the transcripts. The codes were then discussed and the discrepancies were resolved. Similar codes were combined to generate themes.(18) Verbatim are presented to support the findings. We have adhered to the Strengthening the Reporting of Observational studies in Epidemiology (STROBE) and COREQ guidelines to report the study findings. (19)

Ethical issues

Ethics approval was obtained from the Institutional Ethics Committee, Cancer Institute (WIA), Chennai, Tamil Nadu, India and the Ethics Advisory Group of the International Union Against Tuberculosis and Lung Disease, Paris, France. Verbal informed consent was obtained from the participants by telephone. However, the calls were monitored by an individual not associated with the current research.

Patients (Participants) and public involvement

Participants were not involved in the design and conduct of the research, interpretation of results and writing of the manuscript. However, the study results will be disseminated to the participants and public by telephone calls/SMSs and newsletters. Simple short SMSs/messages

will be developed in local language to disseminate the key findings of the study to the study participants. Newsletters in local language will be distributed to the patients and their relatives attending the Cancer Institute where the PI works.

Data sharing statement

Additional data could be made available upon reasonable request at suren.psy@gmail.com

RESULTS

I. Quantitative Component:

Of the 2909 tobacco users, only 724 (24.9%) could be contacted by telephone, of whom 555 (76.7%) consented for the interview. Of those consented, 210 (37.8%) were current tobacco non-users, while 337 (60.7%) were current tobacco users, remaining 08 (1.5%) had missing information. Of those who could not be contacted, the reasons for failing to contact were phone number not recorded (n=738, 33.8%), did not respond (n=109, 5.0%), expired (n=42, 1.9%) and other reasons (n=1296, 59.3%) such as number switched off, incorrect number, not reachable, not a valid number. Among those contacted and consented, 403 (72.6%) have made at least one attempt to quit, of whom 210 (52%) successfully quit, 193 (48%) made a failed quit attempt. Among current tobacco users, 115 (34.1%) did not make any quit attempt and 193 (57.3%) made a failed quit attempt. (**Figure 1b**)

Socio-demographic and characteristics of tobacco use of the respondents are presented in **Table 1**. Most of the respondents (511, 92%) were males. About 60.9% (n=338) were daily wage workers (who do not have a fixed occupation/salary but earn wages on a daily basis) followed by salaried individuals (government or private jobs i.e. those working in the private sector) and 44.3% (n=246) were educated upto secondary level. Majority of the respondents (243, 71.9%) were smokers. **Table 2** compares the socio-demographic characteristics between those contacted versus those who could not be contacted by telephone. Significant difference in educational status was found between the groups (p =0.008).

Majority of the successful quitters reported that they had quit tobacco usage by their own will and determination (110, 50.7%), followed by advice from family (32, 14.7%) and advice from doctors (26, 12.0%). The least sought methods of cessation were counseling (03, 1.4%) and substitution (03, 1.4%) (**Table 3**).

For peer review only - http://bmjopen.bmj.com/site/about/guidelines.xhtml

Significant association between current tobacco use and using smoking form of tobacco products (aRR=1.2, 95% CI: 1.1-1.4) and with exposure to smoke at home, which is a proxy indicator for smoking policy at home (aRR=1.2, 95% CI: 1.1-1.3) was noted (**Table 4**).

II. Qualitative Component:

A total of 26 IDIs were conducted. The socio-demographic details of the participants are given in Table 5. Majority of them were males (22, 84.6%), belonging to the age group 45-59 years (12, 46.2%) and were daily wage laborers (15, 57.7%). The results of the thematic analysis were categorised as: i) barriers, and ii) enablers of tobacco quitting which were further divided into three types: a) intrinsic, b) extrinsic, and c) support system. The themes emerged are presented as a thematic diagram (**Figures 2 and 3**). The details of the themes, sub-themes and verbatim quotes are presented in Table 6.

i. Barriers to quitting

a. Intrinsic factors

Tobacco dependence

Some current smokers talked about the ways in which smoking helped them 'cope' with adverse situations in life, such as giving comfort and relaxation at times of difficulties and thoughts to help manage personal tensions, work life problems and health issues. Consumption of tobacco allegedly helped the respondents to alleviate their pain or stress and improve digestion.

"*Some or the other tension keeps happening. Some problem keeps occurring. At that time, when you smoke it is relaxing, feels good. Smoking one cigarette reduces anger*" *(46, Male)*

No perceived health effects

Some respondents were unaware of the consequences of long-term usage of tobacco while using it spontaneously without any specific intention.

"Health will be affected. We will become weak and have heavy breathing. But I do not do deep inhaling. I smoke very lightly and throw it away. So I think I don't have much effects" (67, Male)

Craving: withdrawal symptoms

Respondents have reported strong urge to smoke and withdrawal symptoms such as nausea, vomiting, tingling sensation in mouth, headache and craving during the evenings after quitting.

For peer review only

b. Extrinsic factors

Availability of tobacco products

Many respondents opined that widespread availability of tobacco products makes it difficult to withhold them from usage.

"We are using because they are selling it. If they do not sell we won't use it" (36, Male)

Social/peer influence

Some participants expressed that the offering of cigarettes from friends and relatives was the main reason for their failure to quit.

"Even if we stay at home wanting to stay away, when other people use, we get the craving. When others use and when they say smoke once nothing will happen, we get the urge" (46, male)

c. Support system

Lack of professional help

Some respondents have cited lack of professional help in terms of counselling or advice as a barrier to quit as they are not confident enough to do it on their own.

"I am unable to do it on my own. I think counselling or any sort of support would help. If possible, you can try to shut down the tobacco companies" (45, male)

ii. Enablers/motivators for quitting

a. Extrinsic factors

Adverse health effects

Recognition of the harms of tobacco to personal health and that of others, especially children in the family was reported to be a motivator for change.

"It is a bad habit, it causes many diseases, children do not like the habit. It is evident that our smoking habit affects others, it affects our health also. It can affect our health, cause cough, cold and cancer" (65, Male)

Responsible parents

Some men spoke of their concerns about the harms from second hand smoke (SHS) and wanted to protect their children and family.

b. Intrinsic factors

Harm to social image

For peer review only

Upholding social image was considered as one of the key component of enablers which helped in successful quit attempt.

"When we smoke around women in a bus stop, they frown. They cover their face with a handkerchief while I smoke. I feel bad. How much ever we act decent, the respect for people who smoke is always less. People don't respect those who smoke" (56, Male)

<u>*Benefits of quitting*</u>

Respondents found many advantages in quitting tobacco use such as being approved by their family, feeling contented from people's approval, financial benefits, and improved health.

"*I was not able to eat much while using tobacco. Now I don't have any such feeling. Since I have quit, I am able to eat good amount of food. I don't have teeth stains and mouth ulcerations*" *(65, Male)*

c. Support system

<u>*Support from family*</u>

Support from children and spouse has been one of the positive reinforces which enabled successful quit attempts.

"My wife, son and friend were against this habit. So I decided to quit. Purely my decision and my wife's support" (65, Male)

<u>*Support from a past quitter*</u>

Support from successful quitters has helped the respondents to quit tobacco use.

"*My close friend had quit and he was supportive. He said it was good that I quit tobacco.*" (39, Male)

<u>*Health advice by doctor*</u>

Advice by doctors also prompted many to quit the habit, especially those who already have adverse physical effects of tobacco.

"*The doctor said, if I continue smoking I might die early. He advised me to quit and he said that all my internal parts have been affected to some extent. He also said that if I continue, I might get TB and other diseases. After that I felt that I should definitely quit*" *(56, Male)*

<u>*Use of substitutes*</u>

Respondents used substitutes such as chocolate, bubble gum, tulsi (*basil*) leaves to overcome craving and sustain the quit attempt.

DISCUSSION

For peer review only

This is the first such study to attempt a follow up of participants of a survey done three years before by telephone calls to understand their current tobacco use status and whether they have made any quit attempt. Only one-fourth of the respondents could be contacted by telephone. This mixed methods assessment among tobacco users of TNTS cohort found that of those contacted and consented for telephone interview, one-third of them have successfully quit tobacco in the last three years and currently are non-tobacco users. Nearly three-quarters have made any quit attempt, of whom half of them could sustain the quit attempt. The qualitative part of the study identified the reasons for failure to quit and the enablers for quitting. The key findings of the study are discussed below.

Unsurprisingly, the study reported poor response rate to a telephone survey. Only one out of four respondents could be contacted. Although telephone surveys have been used widely in public health research and market research, there are concerns regarding poor response rate both due to failure to contact and refusal to participate once contacted. A major reason for poor response rate in this study could be the fact that the contact details of the study participants were collected nearly 3 years ago when the TNTS was conducted. It is highly likely that participants would have changed their numbers which is quite common these days due to cut-throat competition in the telecom market and attractive offers by different network providers. Calls could not be made in a substantial proportion of cases, despite having a telephone number probably due to network issues, improper recording of phone number, tendency of people to switch between networks or possess more than one mobile number etc. Telephone number was not recorded in one-fourth of the respondents, meaning they either did not have any contact number/mobile phone or did not want to share the number or the number was not recorded. These considerations should be weighed in before planning any telephone survey. Moreover, different populations might have different challenges with respect to the use of telephone/mobile phone-based surveys, which needs to understood before planning such surveys. Although telephone surveys yield poor response rates compared to household surveys which have response rates >90%, logistically telephone surveys are preferred.

A study by Boland et al. found poor response rate as low as 17.7% in telephone surveys similar to the present study.(20) In a community based telephone survey in the USA, response rate was 37%.(21) Another study in India in 2006 using telephone survey as a method of data collection yielded a high response rate of 94%. This was probably because it was a landline telephone-based survey and during those times landline numbers did not change frequently. The study

was also done in a limited geographical area in urban location covering 50 households.(22) Based on the study experience and existing literature, we suggest additional strategies such as multi-modal data collection approaches instead of using single method, incentivisation and careful interviewer selection to improve response rate. In this study, the interviewer was a trained staff and part of a call centre of a project routinely involved in making telephone calls to project participants, native of Tamil Nadu (study area) and fluent in the local language (*Tamil)*. However, nearly one-fourth of those who were contacted did not give consent for the interview, which requires additional intervention to improve participation. One such intervention was tried in Australia which concluded that mailing a postcard prior to the first telephone contact increases participation rate.(23)

One-third of the tobacco users have quit tobacco in the last three years and the remaining continue to use tobacco. This is an encouraging finding considering the poor quit rates of 5-10% across several studies. (7,8,24) However, this was self-reported and there was no objective way of assessing this response. A systematic review has shown trends of underestimation when smoking prevalence is based on self-report compared to cotinine-assessed smoking status.(25)

Nicotine addiction has been established the biggest cause of failure in smoking cessation. Tobacco dependence expressed in terms of craving for tobacco products, withdrawal symptoms, psychological dependence and habit forming emerged as the most important barriers to quitting in this study. These factors have specific management implications stressing the need for offering evidence-based tobacco cessation support including medications in line with the MPOWER strategy. The use of smoking cessation aids in our setting has been low similar to the findings of the present study. A national survey in India revealed that nearly 90% of former smokers quit without any professional aid.(26) Participants are reluctant to receive professional help and prefer to 'quit' by themselves. Few of the respondents also reported that quitting was difficult without support and were unaware of the availability of cessation aids. Evidence based tobacco cessation methods should be available and accessible to all through a primary care delivery model. People should be made aware of these services and their role in quitting tobacco and sustaining it.

Peer influence was a major barrier to quitting tobacco as reported in other studies as well.(27–29) Offering cigarettes/tobacco to one another is perceived as a sign of friendship and this

culture serves as an impediment to smoking cessation. People need to be taught methods of rejecting the offer and that declining an offer of a cigarette/tobacco is not seen to be rude.

Most of the respondents reported symptoms of tobacco withdrawal during the initial phase of quitting. At the same time, unanticipated benefits such as a feeling of wellbeing both physically and psychologically, personal satisfaction, improved social relationships, encouragement from the family were also reported, and these benefits were 'self-reinforcing' in helping them to maintain their quit status. Thus, besides the health benefits, the collateral social, economic and psychological gains should also be conveyed to those who are interested in quitting tobacco as part of the counselling package.

The study found that tobacco users with a smoke free policy at home were more likely to quit tobacco. This implies that smoke-free homes influence norms within the family around tobacco use. This inference could also be extended to other public places, thereby generating additional evidence for stricter implementation of smoke-free legislations in all public places.

The study investigators who conducted the IDIs are experienced qualitative researchers with strong interpersonal skills, which is essential in the context of telephone interview to establish rapport quickly and conduct interviews in a conversational manner. These skills helped the interviewer to work through tense and awkward moments that arose during the telephone interaction. Preparation of interviews was also done through mock trainings to handle any situation. The interviewers who work in a cancer care centre were not related to the participants nor were they involved in provision of their care directly or indirectly.

The major strength of the study is that this is the first such attempt to reach out to tobacco users identified in the TNTS 2015-16 after 3 years by a telephone survey. This novel method of survey gave useful insights into the utility of telephone surveys in the Indian context and also provided understanding related to quit attempts and successful quit rates in a large cohort of tobacco users.

The study had two key limitations. The major limitation was the poor response rate of the telephone survey opted due to resource limitation which might have introduced responder bias. However, the baseline characteristics of those who were contacted versus those who could not be contacted by telephone were similar except educational status, suggesting that the results could be generalised to the entire cohort. Secondly, there was no objective means of verifying

the responses received by telephone survey. However, we feel that the social desirability bias is likely to be less in a telephone conversation due to lack of face-to-face interaction.

Conclusion

Nearly two-thirds of the tobacco users have continued using it in the last 3 years. Lack of professional help and tobacco dependence were the major barriers to quitting which warrant decentralised evidence-based cessation interventions. There is evidence for the role of peer-led interventions involving family, peers and other tobacco users in quitting which could be incorporated into cessation interventions.

Recommendations

Future research can consider on-field follow-up of tobacco users, as it could yield higher response rates than telephone follow-up. Research to increase response rates in a telephone survey can also be done. Considering the number of tobacco users who have quit or expressed their willingness to quit by their own self and determination, it is high time to develop interventions involving support system including family, friends and healthcare professionals as these were reported to be major catalysts facilitating quitting of tobacco.

Acknowledgements:

This research was conducted through the Structured Operational Research and Training Initiative (SORT IT), a global partnership led by the Special Programme for Research and Training in Tropical Diseases at the World Health Organization (WHO/TDR). The model is based on a course developed jointly by the International Union Against Tuberculosis and Lung Disease (The Union) and Medécins sans Frontières (MSF/Doctors Without Borders). The specific SORT IT programme which resulted in this publication was jointly developed and implemented by: The Union South-East Asia Office, New Delhi, India; the Centre for Operational Research, The Union, Paris, France; Medécins sans Frontières (MSF/Doctors Without Borders), India; Department of Preventive and Social Medicine, Jawaharlal Institute of Postgraduate Medical Education and Research, Puducherry, India; Department of Community Medicine, All India Institute of Medical Sciences, Nagpur, India; Department of Community Medicine, ESIC Medical College and PGIMSR, Bengaluru, India; Department of Community Medicine, Sri Manakula Vinayagar Medical College and Hospital, Puducherry, India; Karuna Trust, Bangalore, India; Public Health Foundation of India, Gurgaon, India; The INCLEN Trust International, New Delhi, India; Indian Council of Medical Research (ICMR),

view only

Department of Health Research, Ministry of Health and Family Welfare, New Delhi, India; Department of Community Medicine, Sri DevrajUrs Medical College, Kolar, India; and Department of Community Medicine, Yenepoya Medical College, Mangalore, India. We would like to thank the recruiters and the telephone interviewers for the successful conduction of the study.

Funding:

The training program, within which this paper was developed, was funded by the Department for International Development (DFID), UK. The funders had no role in study design, data collection and analysis, decision to publish, or preparation of the manuscript.

Competing interests: None declared.

Author contributions:

Conception and design, Protocol development: SV, EGSV, JPT, NK, TA, RS

Data Collection: SV, RS, DP

Data Analysis: SV, JPT, SR, RS

Drafting the paper: SV, JPT, RS, TA, MM, AN

Critical review and final approval: SV, EGSV, JPT, AK, EH, TA, NK, AN

References

1. WHO Report on The Global Tobacco Epidemic 2019. World Health Organisation. Geneva; 2019.
2. Drope J, Schluger NW, editors. The Tobacco Atlas. Sixth Edit. American Cancer Society; 2018.
3. Fong GT, Hammond D, Laux FL, Zanna MP, Cummings KM, Borland R, et al. The near-universal experience of regret among smokers in four countries: findings from the International Tobacco Control Policy Evaluation Survey. Nicotine Tob Res. 2004 Dec;6 Suppl 3:S341-51.
4. Benowitz NL. Nicotine addiction. N Engl J Med. 2010 Jun 17;362(24):2295–303.
5. Vangeli E, Stapleton J, Smit ES, Borland R, West R. Predictors of attempts to stop smoking and their success in adult general population samples: a systematic review.

Addiction. 2011 Dec;106(12):2110–21.
6. U.S. Department of Health and Human Services. How Tobacco Smoke Causes Disease: The Biology and Behavioural Basis for Smoking - Attributable Disease. Rockville, MD; 2010.
7. Hughes JR, Keely J, Naud S. Shape of the relapse curve and long-term abstinence among untreated smokers. Addiction. 2004 Jan;99(1):29–38.
8. Tobacco Use and Dependence: A 2011 Update of Treatments [Internet]. [cited 2018 Nov 16]. Available from: https://www.medscape.org/viewarticle/757167
9. Ministry of Health & Family Welfare, GOI WHO and T. GATS-2 Global Adult Tobacco Survey Fact Sheet India 2016-17.
10. Dandona L, Dandona R, Anil Kumar G, Shukla DK, Paul VK, Balakrishnan K, et al. Nations within a nation: variations in epidemiological transition across the states of India, 1990 -2016 in the Global Burden of Disease Study. Lancet. 2017;390:2437–60.
11. Resource Centre for Tobacco Control, Cancer Institute(WIA). Tamil Nadu Tobacco Survey(TNTS) 2015-16. Chennai, India; 2016.
12. Creswell J, Plano Clark V. Designing and Conducting Mixed Methods Research. 2nd ed. Thousand Oaks, CA: Sage Publications Ltd; 2011.
13. Census. Primary Census Abstracts, Registrar General of India, Ministry of Home Affairs, Government of India. 2011.
14. Indian States by Economic Freedom - StatisticsTimes.com [Internet]. [cited 2018 Nov 16]. Available from: http://statisticstimes.com/economy/indian-states-economic-freedom.php
15. Shewade HD, Vidhubala E, Subramani DP, Lal P, Bhatt N, Sundaramoorthi C, et al. Open access tools for quality-assured and efficient data entry in a large, state-wide tobacco survey in India. Glob Health Action. 2017;
16. E. V, S. DP, C. S, E. HR, Shewade HD, Lal P. An efficient method to conduct large population survey in a low resource setting: Tamil Nadu tobacco survey. Int J Community Med Public Heal. 2017;4(12):4495.
17. Braun V, Clarke V, Braun V, Clarke V. Applied Qualitative Research in Psychology. Appl Qual Res Psychol. 2017;0887(2006).
18. Lincoln YS, Guba EG. Naturalistic inquiry. Sage Publications; 1985. 416 p.
19. Tong A, Sainsbury P, Craig J. Consolidated criteria for reporting qualitative research (COREQ): a 32-item checklist for interviews and focus groups. Int J Qual Heal care. 2007 Dec 16;19(6):349–57.
20. Boland UM, Sweeney M, Scallan E, Harrington M, Staines A. Emerging advantages and drawbacks of telephone surveying in public health research in Ireland and the. 2006;
21. Krueger AB, Stone AA. Assessment of pain: a community-based diary survey in the USA. Lancet. 2008;371(9623):1519–25.
22. Thulasingam M, Cheriyath PK. Telephone Survey as a Method of Data Collection in South India. 2008;33(4):268–70.
23. Iredell H, Shaw T, Howat P, James R, Granich J. Introductory postcards: Do they increase response rate in a telephone survey of older persons? Health Educ Res. 2004;19(2):159–64.
24. Carlson S, Widome R, Fabian L, Luo X, Forster J. Barriers to Quitting Smoking Among Young Adults: The Role of Socioeconomic Status. Am J Heal Promot. 2018 Feb 7;32(2):294–300.
25. Gorber SC, Schofield-Hurwitz S, Hardt J, Levasseur G, Tremblay M. The accuracy of self-reported smoking: A systematic review of the relationship between self-reported and cotinine-assessed smoking status. Nicotine Tob Res. 2009;11(1):12–24.

For peer review only

26. Srivastava S, Malhotra S, Harries AD, Lal P, Arora M. Correlates of tobacco quit attempts and cessation in the adult population of India: Secondary analysis of the Global Adult Tobacco Survey, 2009-10. Vol. 13, BMC Public Health. 2013.
27. Binnal A, Rajesh GR, Ahmed J, Denny C, Nayak SU. Insights into smoking and its cessation among current smokers in india. Asian Pacific J Cancer Prev. 2013;14(5):2811–8.
28. Berkelmans A, Burton D, Page K, Worrall-Carter L. Registered Nurses' smoking behaviours and their attitudes to personal cessation. J Adv Nurs. 2011 Jul;67(7):1580–90.
29. Ransing RS, Patil DB, Desai MB, Modak A. Outcome of tobacco cessation in workplace and clinic settings: A comparative study. J Int Soc Prev Community Dent. 2016 Sep 1;6(5):487–92.

For peer review only

Table 1: Socio-demographic and characteristics of tobacco use among previously identified tobacco users in eleven selected districts during Tamil Nadu Tobacco Survey (TNTS) (2015-16) who completed the follow up survey' 2019 (N=555)

Characteristics	N	(%)
**Current Tobacco Use**		
Yes	338	(60.9)
No	217	(39.1)
**Type of tobacco use**		
Smoking	243	(71.9)
Smokeless	87	(25.7)
Both	8	(2.4)
**Type of tobacco smoke (n =)**		
Cigarette	151	(27.2)
Bidi	121	(21.8)
Cigar	01	(0.2)
**Type of tobacco smokeless (n =)**		
Tobacco chewing alone	08	(1.4)
Tobacco + Pan masala	68	(12.3)
Snuff	06	(4.5)
Others	35	(6.3)

For peer review only

Table 2 Comparison of socio-demographic characteristics among those contacted versus those who could not be contacted by telephone (n=2909)

Characteristics	Contacted by telephone N=555 n(%)	Could not contact by telephone N=2354 n (%)	p-value
**Age**			0.1
18-24	11 (2.0)	61 (2.6)	
25-44	250 (45.0)	1038 (44.1)	
45-64	247 (44.5)	1020 (43.3)	
≥65	47 (8.5)	235 (10.0)	
**Gender**			0.06
Male	511 (91.8)	2092 (90.6)	
Female	44 (8.2)	260 (9.4)	
**Occupation**			0.12
Unemployed: unable to work	11 (2.0)	71 (3.0)	
Unemployed: able to work	12 (2.2)	47 (2.0)	
Homemaker	25 (4.5)	151 (6.4)	
Daily wage	338 (60.9)	1349 (57.3)	
Self-employed	82 (14.8)	296 (12.6)	
Private/Govt. Job	63 (11.4)	299 (12.7)	
Missing	24 (4.3)	141 (6.0)	
**Education**			0.008
No formal school	17 (3.1)	106 (4.5)	
Primary	105 (18.9)	386 (16.4)	
Secondary	246 (44.3)	929 (39.5)	
Higher secondary and above	86 (15.5)	390 (16.6)	
Missing	101 (18.2)	543 (23.0)	
**Intention to quit***			0.1
Yes	338 (60.9)	1522 (64.5)	
No	148 (26.7)	528 (22.6)	
Missing	69 (12.4)	304 (12.9)	
**Exposure to smoke at home***			0.09
Yes	362 (65.2)	1452 (61.7)	
No	185 (33.3)	857 (36.4)	
Missing	8 (1.5)	45 (1.9)	

*from previous TNTS

For peer review only

694 **Table 3 Method of cessation support sought (last attempt) to quit tobacco among those**
695 **who are current non-smokers (n=210)**

**Cessation method**	**N**	**(%)**
Counselling	03	(1.4)
NRT	05	(2.3)
Other medications	16	(7.4)
Substitution	03	(1.4)
Self (No support)	183	(87.1)
Total	210	(100)

696

697

For peer review only

Table 4: Association of socio-demographic and tobacco use related characteristics with current tobacco user status after the TNTS survey among previously identified tobacco users in 11 selected districts who completed the follow up survey 2019

Characteristics	Total, N	Current tobacco user n (%)*	Non tobacco user n (%)	Unadjusted Relative Risk (95% CI)	p-value	Adjusted Relative Risk (95% CI)
**Age**						
18-24	11	5 (45.5)	6 (55.5)	0.9 (0.5-1.9)	0.8	0.8 (1.5-1.8)
25-44	245	151 (61.6)	94 (38.4)	1.3 (0.9-1.7)	0.13	1.3 (0.8-1.7)
45-64	244	158 (64.8)	86 (35.2)	1.3 (1.0-1.8)	0.05	1.3 (0.9-1.6)
≥65	47	23 (48.9)	24 (51.1)	1.0	-	1.0
**Gender**						
Male	509	313 (61.5)	196 (38.5)	1.0 (0.7-1.2)	0.8	-
Female	38	24 (63.1)	14 (36.8)	1.0	-	
**Occupation**						-
Unemployed	23	12 (52.2)	11 (47.8)	0.8 (0.5-1.3)	0.4	
Homemaker	25	14 (56.0)	11 (44.0)	0.9 (0.6-1.4)	0.6	
Daily Wage	334	212 (63.5)	122 (36.5)	1.0 (0.8-1.3)	0.8	
Self-employed	79	49 (62.0)	30 (38.0)	1.0 (0.8-1.3)	0.9	
Private/Govt job	62	38 (61.3)	24 (38.7)	1.0	-	
**Previous tobacco use**						
Smoking	395	251 (63.5)	144 (36.5)	1.2 (1.1-1.4)	0.04	1.2 (1.1-1.4)*
Smokeless	160	87 (54.4)	73 (45.6)	1.0	-	
**Previous intention to quit**						
Yes	144	82 (56.9)	62 (43.1)	1.0	-	1.0
No	336	214 (63.3)	122 (36.3)	0.9 (0.8-1.1)	0.09	0.9 (0.9-1.2)
**Exposure to smoke at home**$^{\beta}$						
Yes	164	113 (68.9)	51 (31.1)	1.2 (1.1-1.4)	0.008	1.2 (1.1-1.3)*
No	313	182 (58.1)	131 (41.9)	1.0	-	1.0

$^{\beta}$captured during TNTS survey *row percentage; education was removed because it had high multi-collinearity with occupation analysis has been adjusted for clustering at the district level.

For peer review only

Table 5 Socio-demographic characteristics of participants of In-depth interviews, 2019

Characteristics	Frequency	Percentage
**Gender**		
Male	22	84.6
Female	4	15.4
**Age**		
18-24	2	7.7
25-44	9	34.6
45-59	12	46.2
≥60	3	11.5
**Occupation**		
Homemaker	3	11.5
Daily wage	15	57.7
Self-employed	5	19.2
Private/Govt. Job	3	11.5
**Education**		
Primary	2	7.7
Secondary	16	61.5
Higher secondary and above	5	19.2
Missing	3	11.5
**Quit attempt**		
Successful attempt	10	38.4
Failed attempt	10	38.4
Did not attempt	6	23.1

For peer review only

Table 6: Themes and sub-themes of enablers and barriers of quitting tobacco and sustaining it with corresponding quotes

	Theme	Sub-theme		Quotes
Enablers	Extrinsic	Adverse health effects: Self & Others		*"It affects everything. It is a bad habit. It is harmful to health. I get cough, cold. All the internal organs are affected because of this. Quitting this is a very good deed"*
		Responsible parents		"*Doctors are saying that it affects the children immediately. All I want is children should not be affected, people at home should respect me and I should not have cough anymore. When my children said quit this, I decided to quit*"
	Intrinsic	Harm to social image		*"People around us used to frown when we are using tobacco next to them. I used to think whether it is such a horrible thing"*
		Benefits of quitting	Immediate effects	*"That is a very satisfying thing for me. I don't have any cough or cold after quitting"*
			Feel happy and satisfied	*"I am feeling good now. Because, I was addicted to a bad habit, but I have quit now. I feel that it's a good thing"*
			Perceived health benefits	*"Used to get cold, cough and would feel suffocated when smoking. Now after quitting, I am able to breathe normally. I am not getting exhausted now. I am able to feel that clearly. I am feeling happy that I quit"*
			Improved social and family relationships	*"I don't have cough. Now I can play with children. Initially I used to have a guilt that I keep coughing while playing with children"*
			Financial gains	*"When I am spending the 30 or 40 rupees from not purchasing cigarettes, for the sake of my children, I feel happy"*

	Support system	Support from family		*"Family was very supportive. They always advise not to drink and not to smoke. Wife fights, daughter fights. It's a problem for everyone"*
		Support from past quitter		*"My friend advised that it would be beneficial to quit and that someone would be motivated to quit after seeing me"*
		Health advice by a doctor		*"Doctor advised me not to use this tobacco. I checked with him because I had burning sensation in the chest. Doctor said that it might be because of the tobacco that I use and advised me to reduce it"*
		Use of substitutes		*"I used to take tobacco after tea. Now as soon as I have tea I keep something in my mouth. I get the craving when I see people using tobacco, but I take vicks tablet at that time"*
Barriers	Intrinsic	Tobacco Dependence	Coping with personal issues	*"I smoke definitely when I am tensed. I smoke two to three cigarettes at a time when I am angry. If people make me angry, I will smoke to relax myself"*
			Pain/ Stress reliever	*"I use it occasionally, when I have toothache. Otherwise I won't. Only for toothache"*
			Improves digestion	*"I smoke only one cigarette after food. I use it for better digestion, that's it"*
		Casual usage: No perceived health effects		*"I will have such effects only if I use tobacco every day. But I use it only when I have toothache. So I don't have any effects"*
		Habitual user		*"What to do… Since it has become a habit for so long, I am unable to quit"*
		Craving: Withdrawal symptoms		*"When I am in the middle of a conversation, at times, I have this craving suddenly and I feel like I have to go immediately. I am unable to control the urge"*

For peer review only

Extrinsic	Availability of tobacco products	*"We are using this because it's available in the shops. Also, when someone smokes and exhales in front us, we get the craving"*
	Social/ Peer influence	*"Even if I stay at home trying to not use tobacco, I would want to use when someone who is using tobacco comes and says, just use it once"*
Support system		*"If I am to quit, I will have to do it on my own will. Counselling or any sort of advice from others will not help in this case. Even when my family advices me, I move away from that place. I can quit, only if I make that decision on my own"*

For peer review only

Figure 1a Standard Operating Procedure for making telephone calls

The telephonic calls were placed by a trained project staff

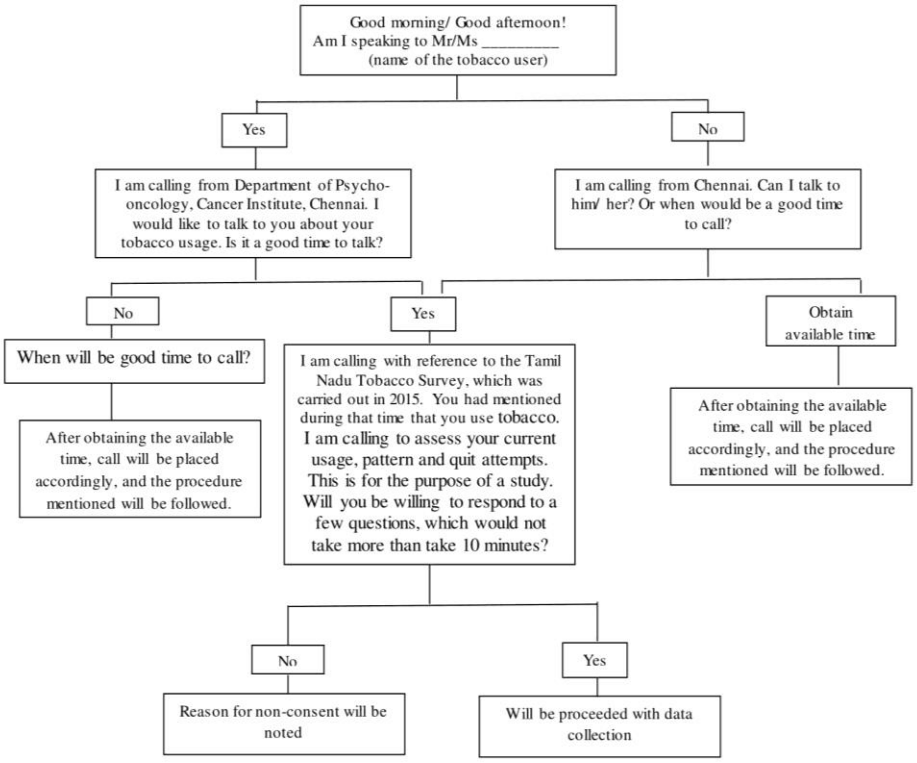

Figure 1b: Flow diagram depicting the status of current tobacco use and the pattern of quit attempts among tobacco users in six selected districts of Tamil Nadu previously identified in the Tamil Nadu Tobacco Survey (TNTS) (2015-16)

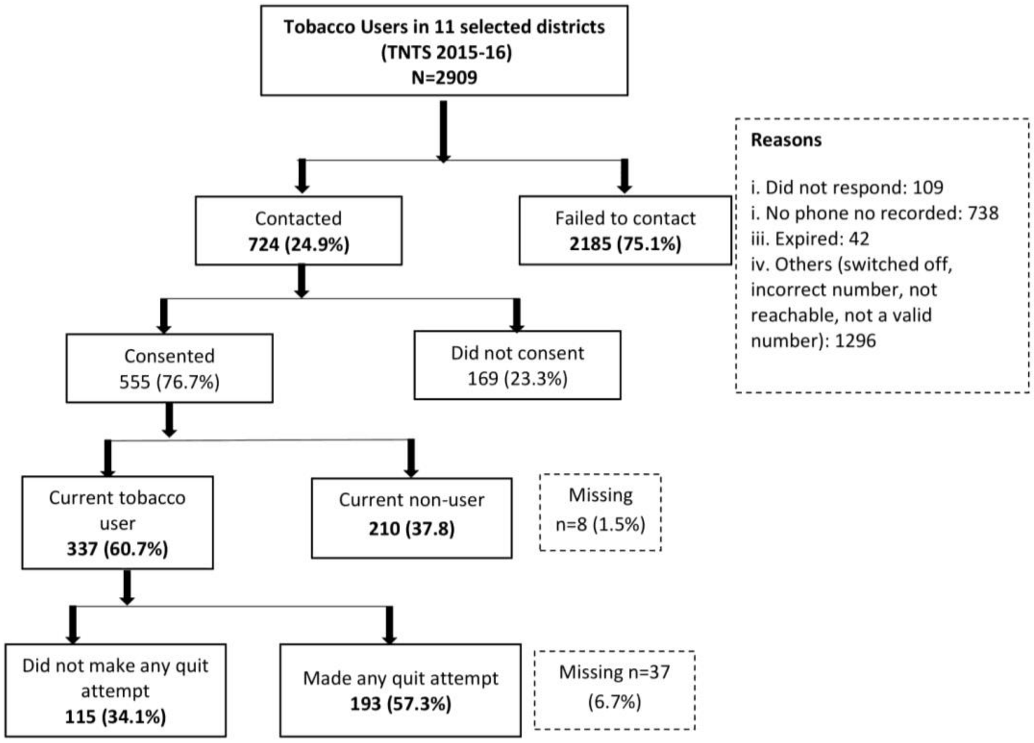

Figure 2 Enablersof quitting tobacco and sustaining it among the tobacco users in three selected districts of Tamil Nadu, 2019

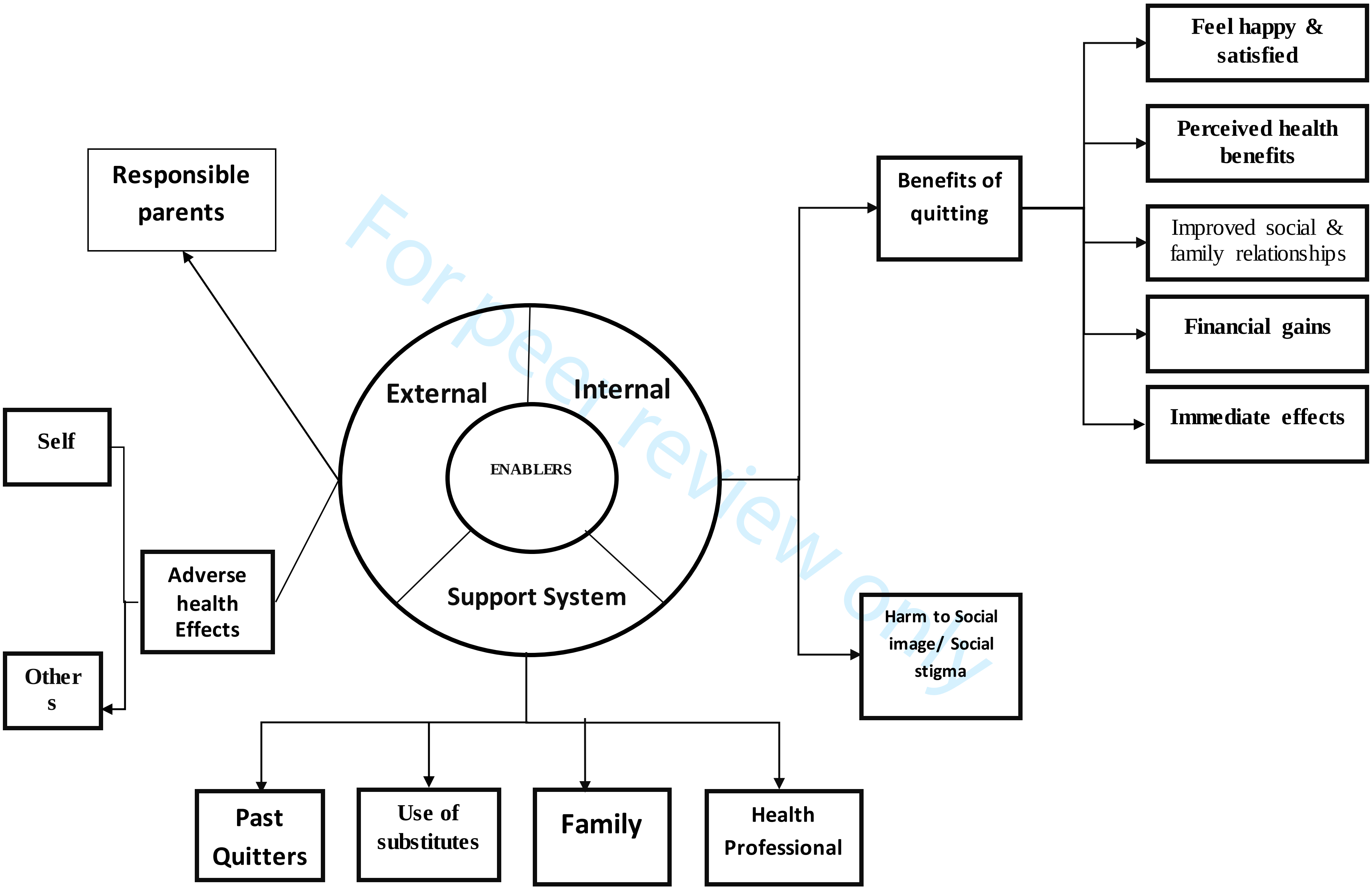

Figure 3 Barriers of quitting tobacco and sustaining it among the tobacco users in three selected districts of Tamil Nadu, 2019

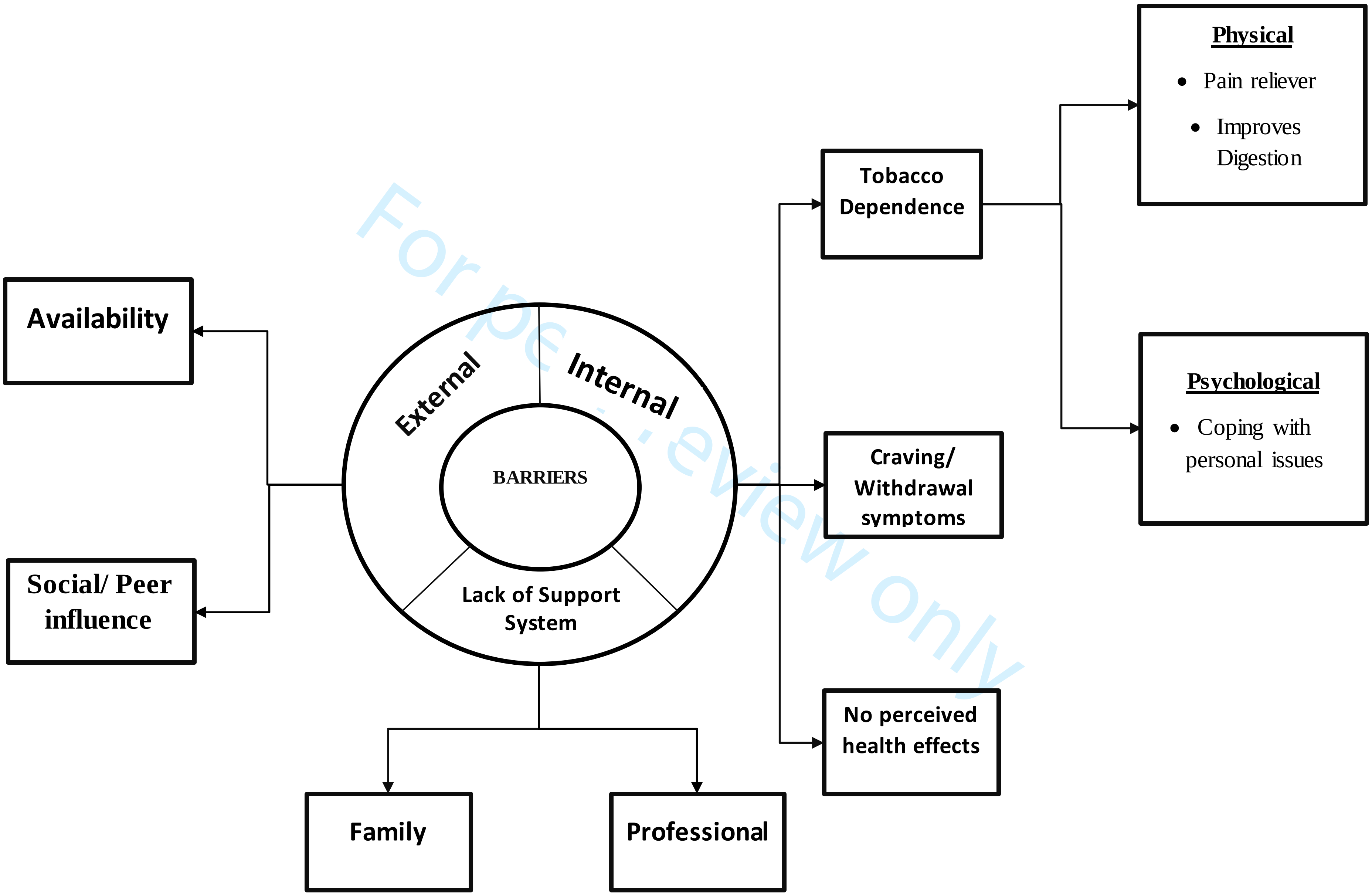

For peer review only - http://bmjopen.bmj.com/site/about/guidelines.xhtml

STROBE Statement—Checklist of items that should be included in reports of ***cohort studies***

	Item No	Recommendation	Page No
**Title and abstract**	1	(*a*) Indicate the study's design with a commonly used term in the title or the abstract	1
		(*b*) Provide in the abstract an informative and balanced summary of what was done and what was found	3
**Introduction**			
Background/rationale	2	Explain the scientific background and rationale for the investigation being reported	4
Objectives	3	State specific objectives, including any prespecified hypotheses	5
**Methods**			
Study design	4	Present key elements of study design early in the paper	6
Setting	5	Describe the setting, locations, and relevant dates, including periods of recruitment, exposure, follow-up, and data collection	6
Participants	6	(*a*) Give the eligibility criteria, and the sources and methods of selection of participants. Describe methods of follow-up	6
		(*b*) For matched studies, give matching criteria and number of exposed and unexposed	
Variables	7	Clearly define all outcomes, exposures, predictors, potential confounders, and effect modifiers. Give diagnostic criteria, if applicable	7
Data sources/ measurement	8*	For each variable of interest, give sources of data and details of methods of assessment (measurement). Describe comparability of assessment methods if there is more than one group	7
Bias	9	Describe any efforts to address potential sources of bias	
Study size	10	Explain how the study size was arrived at	7
Quantitative variables	11	Explain how quantitative variables were handled in the analyses. If applicable, describe which groupings were chosen and why	7
Statistical methods	12	(*a*) Describe all statistical methods, including those used to control for confounding	9
		(*b*) Describe any methods used to examine subgroups and interactions	
		(*c*) Explain how missing data were addressed	
		(*d*) If applicable, explain how loss to follow-up was addressed	
		(*e*) Describe any sensitivity analyses	
**Results**			
Participants	13*	(a) Report numbers of individuals at each stage of study—eg numbers potentially eligible, examined for eligibility, confirmed eligible, included in the study, completing follow-up, and analysed	10
		(b) Give reasons for non-participation at each stage	10
		(c) Consider use of a flow diagram	10
Descriptive data	14*	(a) Give characteristics of study participants (eg demographic, clinical, social) and information on exposures and potential confounders	10
		(b) Indicate number of participants with missing data for each variable of interest	10
		(c) Summarise follow-up time (eg, average and total amount)	10
Outcome data	15*	Report numbers of outcome events or summary measures over time	10

For peer review only

Main results	16	(*a*) Give unadjusted estimates and, if applicable, confounder-adjusted estimates and their precision (eg, 95% confidence interval). Make clear which confounders were adjusted for and why they were included<br>(*b*) Report category boundaries when continuous variables were categorized<br>(*c*) If relevant, consider translating estimates of relative risk into absolute risk for a meaningful time period	11
Other analyses	17	Report other analyses done—eg analyses of subgroups and interactions, and sensitivity analyses	
**Discussion**			
Key results	18	Summarise key results with reference to study objectives	16
Limitations	19	Discuss limitations of the study, taking into account sources of potential bias or imprecision. Discuss both direction and magnitude of any potential bias	16
Interpretation	20	Give a cautious overall interpretation of results considering objectives, limitations, multiplicity of analyses, results from similar studies, and other relevant evidence	16
Generalisability	21	Discuss the generalisability (external validity) of the study results	16
**Other information**			
Funding	22	Give the source of funding and the role of the funders for the present study and, if applicable, for the original study on which the present article is based	17

For peer review only

COREQ (COnsolidated criteria for REporting Qualitative research) Checklist

A checklist of items that should be included in reports of qualitative research. You must report the page number in your manuscript where you consider each of the items listed in this checklist. If you have not included this information, either revise your manuscript accordingly before submitting or note N/A.

Topic	Item No.	Guide Questions/Description	Reported on Page No.
**Domain 1: Research team and reflexivity**			
Personal characteristics			
Interviewer/facilitator	1	Which author/s conducted the interview or focus group?	8
Credentials	2	What were the researcher's credentials? E.g. PhD, MD	M.Phil., PhD & M.Phil
Occupation	3	What was their occupation at the time of the study?	1
Gender	4	Was the researcher male or female?	
Experience and training	5	What experience or training did the researcher have?	8
Relationship with participants			
Relationship established	6	Was a relationship established prior to study commencement?	6
Participant knowledge of the interviewer	7	What did the participants know about the researcher? e.g. personal goals, reasons for doing the research	8
Interviewer characteristics	8	What characteristics were reported about the inter viewer/facilitator? e.g. Bias, assumptions, reasons and interests in the research topic	8
**Domain 2: Study design**			
Theoretical framework			
Methodological orientation and Theory	9	What methodological orientation was stated to underpin the study? e.g. grounded theory, discourse analysis, ethnography, phenomenology, content analysis	9
Participant selection			
Sampling	10	How were participants selected? e.g. purposive, convenience, consecutive, snowball	6
Method of approach	11	How were participants approached? e.g. face-to-face, telephone, mail, email	8
Sample size	12	How many participants were in the study?	8
Non-participation	13	How many people refused to participate or dropped out? Reasons?	10
Setting			
Setting of data collection	14	Where was the data collected? e.g. home, clinic, workplace	8
Presence of non-participants	15	Was anyone else present besides the participants and researchers?	8
Description of sample	16	What are the important characteristics of the sample? e.g. demographic data, date	10
Data collection			
Interview guide	17	Were questions, prompts, guides provided by the authors? Was it pilot tested?	8
Repeat interviews	18	Were repeat inter views carried out? If yes, how many?	
Audio/visual recording	19	Did the research use audio or visual recording to collect the data?	8
Field notes	20	Were field notes made during and/or after the inter view or focus group?	8
Duration	21	What was the duration of the inter views or focus group?	8

For peer review only

For peer review only - http://bmjopen.bmj.com/site/about/guidelines.xhtml

Data saturation	22	Was data saturation discussed?	8
Transcripts returned	23	Were transcripts returned to participants for comment and/or	8

Topic	Item No.	Guide Questions/Description	Reported on Page No.
		correction?	
**Domain 3: analysis and findings**			
Data analysis			
Number of data coders	24	How many data coders coded the data?	9
Description of the coding tree	25	Did authors provide a description of the coding tree?	11
Derivation of themes	26	Were themes identified in advance or derived from the data?	9
Software	27	What software, if applicable, was used to manage the data?	9
Participant checking	28	Did participants provide feedback on the findings?	8
Reporting			
Quotations presented	29	Were participant quotations presented to illustrate the themes/findings? Was each quotation identified? e.g. participant number	11
Data and findings consistent	30	Was there consistency between the data presented and the findings?	15
Clarity of major themes	31	Were major themes clearly presented in the findings?	11
Clarity of minor themes	32	Is there a description of diverse cases or discussion of minor themes?	11

For peer review only

Developed from: Tong A, Sainsbury P, Craig J. Consolidated criteria for reporting qualitative research (COREQ): a 32-item checklist for interviews and focus groups. *International Journal for Quality in Health Care*. 2007. Volume 19, Number 6: pp. 349 – 357

Once you have completed this checklist, please save a copy and upload it as part of your submission. DO NOT include this checklist as part of the main manuscript document. It must be uploaded as a separate file.

For peer review only

BMJ Open

Quit attempts amongst tobacco users identified in the Tamil Nadu Tobacco Survey of 2015-16: A 3 year follow-up mixed methods study

Journal:	*BMJ Open*
Manuscript ID	bmjopen-2019-034607.R2
Article Type:	Original research
Date Submitted by the Author:	29-May-2020
Complete List of Authors:	Veeraiah, Surendran; Cancer Institute-WIA, Psycho-oncology<br>Elangovan, Vidhubala; Fenivi Research Solutions<br>Tripathy, Jaya; All India Institute of Medical Sciences - Nagpur<br>Krishnamurthy, Arvind; Cancer Institute-WIA<br>Anand , T ; Indian Council of Medical Research<br>M, Mahendra; Sri Devraj Urs Medical College<br>Sudhakar, Revathy; Cancer Institute-WIA, Psycho-oncology<br>K, Niraimathi; Fenivi Research Solutions<br>Subramani, Divyarajprabhakar; Cancer Institute-WIA, Psycho-oncology<br>Rajaraman, Swaminathan; Cancer Institute-WIA<br>Elluswami, Hemanth Raj; Cancer Institute-WIA<br>Nirgude, Abhay; Yenepoya Medical College Hospital
**Primary Subject Heading**:	Public health
Secondary Subject Heading:	Smoking and tobacco, Epidemiology
Keywords:	PUBLIC HEALTH, EPIDEMIOLOGY, PREVENTIVE MEDICINE

SCHOLARONE™
Manuscripts

I, the Submitting Author has the right to grant and does grant on behalf of all authors of the Work (as defined in the below author licence), an exclusive licence and/or a non-exclusive licence for contributions from authors who are: i) UK Crown employees; ii) where BMJ has agreed a CC-BY licence shall apply, and/or iii) in accordance with the terms applicable for US Federal Government officers or employees acting as part of their official duties; on a worldwide, perpetual, irrevocable, royalty-free basis to BMJ Publishing Group Ltd ("BMJ") its licensees and where the relevant Journal is co-owned by BMJ to the co-owners of the Journal, to publish the Work in this journal and any other BMJ products and to exploit all rights, as set out in our licence.

The Submitting Author accepts and understands that any supply made under these terms is made by BMJ to the Submitting Author unless you are acting as an employee on behalf of your employer or a postgraduate student of an affiliated institution which is paying any applicable article publishing charge ("APC") for Open Access articles. Where the Submitting Author wishes to make the Work available on an Open Access basis (and intends to pay the relevant APC), the terms of reuse of such Open Access shall be governed by a Creative Commons licence – details of these licences and which Creative Commons licence will apply to this Work are set out in our licence referred to above.

Other than as permitted in any relevant BMJ Author's Self Archiving Policies, I confirm this Work has not been accepted for publication elsewhere, is not being considered for publication elsewhere and does not duplicate material already published. I confirm all authors consent to publication of this Work and authorise the granting of this licence.

For peer review only

Title Page

Quit attempts amongst tobacco users identified in the Tamil Nadu Tobacco Survey of 2015-16: A 3 year follow-up mixed methods study

Short running title: Successful tobacco quit attempts: enablers and barriers

Authors and affiliations

Surendran Veeraiah[1], E Grace Sahaya Vidhubala[2], Jaya Prasad Tripathy[3], Arvind Krishnamurthy[1], Tanu Anand[4], Mahendra M[5], Revathy Sudhakar[1], Niraimathi K[2], Divyaraj Prabhakar[1], Swaminathan R[1], E Hemanth Raj[1], Abhay Nirgude[6]

1 Cancer Institute (WIA), Chennai, India

2 Fenivi Research Solutions, Chennai, India

3 All India Institute of Medical Sciences, Nagpur, India

4 Indian Council of Medical Research, Department of Health Research, Ministry of Health and Family Welfare, New Delhi

5 Sri Devraj Urs Medical College, Kolar, Karnataka

6 Yenepoya Medical College, Mangalore, India

Details of the corresponding author

Surendran Veeraiah

Associate Professor and Head

Department of Psycho-oncology and Resource Centre for Tobacco Control

Cancer Institute (WIA),

38, Sardar Patel Road, Adyar, Chennai,

Tamil Nadu, India. PIN 600036

suren.psy@gmail.com

v.surendran@cancerinstitutewia.org

For peer review only

Word Count: Manuscript: 3941; Abstract: 278

Number of tables: 5

Number of figures: 3

Number of references: 26

Keywords- Quit attempt, Tobacco use, Enablers & Barriers, SORT-IT, telephone survey

For peer review only

Abstract

Objectives

To determine current tobacco use in 2018-19, quit attempts made and to explore the enablers and barriers in quitting tobacco among tobacco users identified in the Tamil Nadu Tobacco Survey (TNTS) in 2015-16.

Setting

TNTS was conducted in 2015-16 throughout the state of TN in India covering 111363 individuals. Tobacco prevalence was found to be 5.2% (n=5208)

Participants

All tobacco users in eleven districts of TN identified by TNTS (n=2909) were tracked after three years by telephone. In-depth interviews (n=26) were conducted in a sub-sample to understand the enablers and barriers in quitting.

Primary and Secondary Outcomes

Current tobacco use status, any quit attempt and successful quit rate were the primary outcomes, while barriers and enablers in quitting were considered as secondary outcomes.

Results

Among the 2909 tobacco users identified in TNTS 2015-16, only 724 (24.9%) could be contacted by telephone, of which 555 (76.7%) consented. Of those who consented, 210 (37.8%) were currently not using tobacco (i.e. successfully quit) and 337 (60.7%) continued to use any form of tobacco. Of current tobacco users, 115 (34.1%) never made any quit attempt and 193 (57.3.8%) have made any attempt to quit. Those using smoking form of tobacco products (aRR=1.2, 95% CI: 1.1-1.4) and exposure to smoke at home (aRR=1.2, 95% CI: 1.1-1.3) were found to be positively associated with continued tobacco use (failed or no quit attempt). Support from family and perceived health benefits are key enablers, while peer influence, high dependence and lack of professional help are some of the barriers to quitting.

Conclusion

Two-thirds of the tobacco users continue to use tobacco in the last 3 years. While tobacco users are well aware of the ill-effects of tobacco, various intrinsic and extrinsic factors play a major role as a facilitator and lack of the same act as a barrier to quit.

Strengths and limitations of this study

- This is the first such study that we are aware of, to attempt a follow-up of tobacco users identified in previous survey to understand their current tobacco use status and quit attempts.
- The study involved telephone survey to contact the tobacco users
- The mixed-methods design enabled estimation of quit rates and understanding the enablers and barriers in quitting tobacco.
- A major limitation of this study was the poor response rate of the telephonic survey which might have introduced responder bias.
- There was no objective means of verifying the responses received by telephone.

INTRODUCTION

The tobacco epidemic continues to be a major public health concern with nearly 1.4 billion tobacco users worldwide. It is one of the most important preventable causes of premature death in the world claiming more than 8 million lives each year.(1,2)

To address the growing tobacco menace, the World Health Organization Framework Convention on Tobacco Control (WHO FCTC) came into force in 2005. This international treaty has been ratified by 181 countries, and provides a roadmap for the countries to adopt and implement tobacco control measures. Article 14 of WHO FCTC mentions the dissemination of comprehensive guidelines based on scientific evidence to promote tobacco cessation. To assist in country-level implementation of the WHO FCTC, WHO also introduced a package of six technical measures termed as the MPOWER strategy, where ‘O’ stands for ‘offer help to quit tobacco use’ which is one of the key components of this strategy.

It is beyond any doubt that quitting tobacco is one of the most effective ways of saving lives and improving overall well-being. Majority of the smokers regret ever starting to smoke and

For peer review only

want to quit.(3) However, quitting smoking remains difficult primarily because of the addictiveness of nicotine in tobacco, along with other social and contextual factors.(4–6)It is reported that only about 3-5% of unassisted quit attempts are successful.(7,8)

In India, the prevalence of tobacco use in any form is 29% of all adults (42% of men and 14% of women).(9) Tobacco use contributes to nearly 10% of all deaths in the country with more than 1 million deaths in 2016.(10) According to the Global Adult Tobacco Survey 2016-17 (GATS) in India, more than half of current tobacco users were planning or thinking of quitting tobacco use.(9) However, we do not know how many of them actually made a quit attempt or went on to become a successful quitter. Several other large nationally representative cross-sectional studies such as GATS, National Health Family Surveys etc. have examined tobacco prevalence. However, these surveys are cross-sectional in nature with limited cohort-wise assessment of tobacco users and their quitting behaviour over a period of time.

A cross-sectional household tobacco survey, Tamil Nadu Tobacco Survey (TNTS), was conducted in 2015-16 in the state of Tamil Nadu, South India by the Cancer Institute (Women's India Association), Chennai, India to provide reliable state and district-wise estimates of tobacco use.(11) The survey covered nearly 100 000adults (>15 years) in all 32 districts across the state. The results of the survey showed that 5.2% were current tobacco users and about one in every five tobacco users reported to have intention to quit tobacco use in the next one month. But how many of them actually quit and how many of those who made a quit attempt were successful, is unknown.

In order to answer these questions, we did a follow-up of those who were identified as tobacco users in the TNTS three years post-survey by telephone to understand their current tobacco use status and any quit attempts made in the last three years. After quantitatively assessing tobacco use status, quit rates and quit attempts among previous tobacco users, it is also useful to understand the enablers that motivated and barriers they faced in quitting or attempting to quit tobacco through a qualitative approach. This will help design a tailored package of cessation and counselling intervention. Hence, a sequential explanatory mixed-method design was adopted for this study wherein the sample for qualitative study was a subset of the quantitative sample.

The specific objectives of the study were:

For peer review only

1. Among the tobacco users previously identified in the TNTS in 2015-16, determine the number and proportion who could be contacted through a telephone survey in 2018-19 and compare their characteristics with those who could not be contacted
2. Among those contacted by telephone in 2018-19, determine the number and proportion who i) continue to use tobacco (smoking and/or smokeless) i.e. failed or no quit attempt, ii) made a successful quit attempt, iii) made any quit attempt,
3. Explore the barriers and enablers in making and sustaining a quit attempt.

METHODS

Study Design

This study employed a sequential explanatory QUAN-QUAL mixed-methods design with a cohort study design as the quantitative component followed by a descriptive qualitative component. (12) The quantitative cohort study was a follow-up of assessment of tobacco users identified during the TNTS in 2015-16 to assess their current tobacco use and quit attempts. Following the quantitative telephone survey, the participants were categorised into three groups based on the quit attempt made and the success of the attempt. The qualitative sample was chosen from these groups proportionate to the size of the groups. Therefore, a sequential design was opted in which the qualitative component followed the quantitative one.

Setting

General setting:

In order to tackle the burden of tobacco use in the country, the Ministry of Health and Family Welfare launched a network of 19 tobacco cessation clinics (TCCs) in India in 2002 with the support from WHO. These clinics offer a wide variety of behavioural (brief advice, 5A's and 5R's, individual/group counselling) and pharmacological interventions (Nicotine Replacement Therapy: nicotine patch, gum, inhaler, spray and non-nicotine replacement therapy: bupropion, varenicline) for tobacco cessation free of cost. A combination of behavioural support and pharmacotherapy is generally considered the best approach for treating tobacco dependence. Subsequently, the National Tobacco Control Programme was launched in 2007-08 to be implemented by Tobacco Control Cells at the national, state and district level. Under this program, there is also a provision of setting up Tobacco Cessation Services at the district level. India has also launched quitline (toll free helpline service) and Cessation program wherein tobacco users can register to receive tailored cessation advice via mobile messages.

For peer review only

Tamil Nadu (TN) is the sixth largest state by population with about 72 million people.(13) It has 32 administrative districts. With nearly half of the population residing in urban areas, it has a high literacy rate of 80% (14).In TN, according to GATS 2, nearly 20% use tobacco in any form, of whom 9.5% are smokers, 9.5% are smokeless tobacco users and remaining 1% use both.(9)

Specific setting:

Tamil Nadu Tobacco Survey (TNTS) 2015-16

The TNTS identified 111,363 eligible individuals aged 15 years and above, from 32,945 households across all 32 districts in TN. Of these, 99,825 individuals contacted door to door, responded, with the response rate being 89.2%. All these individuals were assessed for tobacco use, exposure to second hand smoke, quit behaviour, impact of pictorial warnings and other tobacco control legislations.

Survey sampling methodology

Under TNTS, each of the 32 districts was divided into urban and rural areas, whereas Chennai city was divided into 15 zones, each zone further sub-divided into slum and non-slum. The estimated sample was divided among all urban and rural areas of districts, slums and non-slum areas of zones in Chennai city using Probability Proportional to Size sampling [6]. Data were collected during 2015-2016. The details of the survey methodology are given elsewhere.(11,15,16)

Study population/Sampling frame

The study population for both the quantitative and qualitative component included all the identified tobacco users (n=5208) from the TNTS 2015-16. The quantitative sample was recruited by a telephone survey. The qualitative sample (n=26) is a subset of the quantitative sample.

Sample size

Assuming that about 6% of tobacco users make a successful quit attempt, with 2% absolute precision and 80% power, sample size was calculated to be 610. Assuming 33% response rate from our previous experience (this being a telephonic survey), the final sample size was

For peer review only

estimated to be 2025. These participants were recruited from the original TNTS survey conducted in 2015-16 by telephonic survey.

Data variables, sources of data and data collection

Quantitative

Data were collected from two sources: a) TNTS database (already collected in 2015-16) and b) Telephonic survey (conducted in 2018-19). A structured questionnaire was used to collect information by telephone survey with the respondents of the original TNTS survey, with the items broadly covering areas such as current tobacco usage (both smoking and/or smokeless), quit attempt(s) and their duration and their intention to quit. In addition, socio-demographic and tobacco use related variables were extracted from the TNTS. Reported tobacco users of TNTS (N=2909) in 11 districts of TN were contacted through telephone by a team of trained project staff at the Cancer Institute, Chennai. A Standard Operating Procedure (SOP) was prepared and followed for telephone survey (**Figure 1**)**.** Each study participant was contacted a maximum of three times at an interval of 30 minutes in a day. After two calls, a standardized text message was sent stating the details of the caller and the purpose of the call. Subsequently, the tobacco user was called 30 minutes after the text message. This process was repeated again after 7 days (if no contact was made in the previous attempt) before labeling it as an unsuccessful contact. Response to each call was recorded by the project staff using a separate sheet as: no response, disconnected the call, number not reachable, number invalid, refused to share information, busy schedule, responded to the call and so on. Respondents who were contacted and verbally consented to participate were briefed about the purpose of the call. The questions were administered over telephone and the responses were recorded on a structured questionnaire. The telephone calls were not recorded as it might affect the responses of the participant. However, the telephone survey was monitored by an individual not associated with the current research for interviewer compliance with the protocol described above. Verbal feedback was given continuously to improve and finetune the process.

Qualitative

The Principal Investigator (PI) (Ph.D. in Psychology) and the co-PI (M.Phil.) who are trained in qualitative research methods conducted In-Depth Interviews (IDIs)after obtaining consent of the participants.The IDIs were conducted in regional language (*Tamil*) by telephone using an interview guide with open ended questions related to the quit attempts made, method of

For peer review only

quitting and motivation to quit and barriers/motivators for failed or successful quit attempts. This data was collected separately and not as a part of the quantitative data collection with all the participants, due to time constraint. The interviews were audio recorded (after obtaining consent) and verbatim notes were also taken during the interview. Each interview lasted for around 30 minutes. After the interview was over, the summary of the interviews was read back to the participants to ensure participant validation. Since it was a telephone interview, no incentives were provided for the participants. A total of 8-10 IDIs were planned to be conducted in each district to cover those who made a successful quit attempt, failed attempt and did not made a quit attempt. It was planned to cover both smokers and smokeless tobacco users in the sample.

Operational Definitions (11)

Quit attempt

Any attempt at tobacco cessation that lasts for 1 or more than one day, including both self-attempt as well as attempt with professional help.

Current tobacco users

Tobacco users, who reported using any form of tobacco daily or occasionally for more than one month prior to the interview.

Sampling

Quantitative

All tobacco users identified in TNTS 2015-16 in 11 purposively selected districts namely Chennai, Coimbatore, Kanchipuram, Madurai, Tirunelveli and Tiruvallur, Viluppuram, Pudukkottai, Kanyakumari, Tiruppur and Erode were recruited (n=2909) consecutively. These districts were purposively selected to ensure wider geographical coverage.

Qualitative

The sample for IDIs included a conveniently selected lot of tobacco users identified through TNTS 2015-16, residing in Chennai, Kanchipuram and Thiruvallur Districts. A total of 26 IDIs were conducted. The participants of the telephone survey were divided into three groups: i) those who made a failed quit attempt (n=10), ii) made a successful quit attempt (n=10), and iii) those who did not make any attempt (n=6). Around 6-10 IDIs were conducted in each of the three districts from three groups. Maximum variation sampling was used to include both smokers and smokeless tobacco users from different age groups. Data saturation was practiced using informational redundancy approach (17). Further interviews were discontinued if no new

For peer review only

information was obtained pertaining to the major themes. However, there was inadequate response from the third group where the participants did not make any quit attempt.

Analysis and Statistics

Quantitative

Quantitative data were double entered and validated using EpiData entry (version 3.0) and analysed using EpiData analysis (version 2.2.2.183, EpiData Association, Odense, Denmark) and STATA version 13.0. The key outcome indicators were current tobacco use and quit attempt. Chi-square test was used to find the association between various socio-demographic, tobacco use related variables with the current tobacco use. Binomial regression was used to explore the factors associated with tobacco use. Adjusted Relative Risks (aRRs) with 95% confidence intervals was used to measure the strength of the association.

Qualitative

The audio recorded interviews were transcribed manually in local language, Tamil, by the PI (SV) and the co-PI (RS) as soon the interviews were over. The transcripts were read multiple times by two investigators (SV and RS) before coding. Thematic analysis following the six-phase approach by Braun & Clarke (2006) was undertaken to analyse the transcripts.(18) A hierarchical codebook was developed by two study investigators (SV and RS) by synthesizing codes emerging directly from the transcripts (inductive) and from the topic guides (deductive).The initial coding was done independently by the investigators after going through the transcripts. The codes were then discussed and the discrepancies were resolved. Similar codes were combined to generate themes.(19) Verbatim are presented to support the findings. We have adhered to the Strengthening the Reporting of Observational studies in Epidemiology (STROBE) and COREQ guidelines to report the study findings. (20)

Ethical issues

Ethics approval was obtained from the Institutional Ethics Committee, Cancer Institute (WIA), Chennai, Tamil Nadu, India and the Ethics Advisory Group of the International Union Against Tuberculosis and Lung Disease, Paris, France. Verbal informed consent was obtained from the participants by telephone. However, the calls were monitored by an individual not associated with the current research.

Patients (Participants) and public involvement

Participants were not involved in the design and conduct of the research, interpretation of results and writing of the manuscript. However, the study results will be disseminated to the participants and public by telephone calls/SMSs and newsletters. Simple short SMSs/messages will be developed in local language to disseminate the key findings of the study to the study participants. Newsletters in local language will be distributed to the patients and their relatives attending the Cancer Institute where the PI works.

Data sharing statement

Extra data can be accessed via the Dryad data repository at http://datadryad.org/ with the doi: 10.5061/dryad.gtht76hj5

RESULTS

Of the 2909 tobacco users, only 724 (24.9%) could be contacted by telephone, of whom 555 (76.7%) consented for the interview. Of those consented, 210 (37.8%) were current tobacco non-users, while 337 (60.7%) were current tobacco users, remaining 08 (1.5%) had missing information. Of those who could not be contacted, the reasons for failing to contact were phone number not recorded (n=738, 33.8%), did not respond (n=109, 5.0%), expired (n=42, 1.9%) and other reasons (n=1296, 59.3%) such as number switched off, incorrect number, not reachable, not a valid number. **Figure 1**

Socio-demographic and characteristics of tobacco use of the respondents are presented in **Table 1**. Most of the respondents (511, 92%) were males. About 60.9% (n=338) were daily wage workers (who do not have a fixed occupation/salary but earn wages on a daily basis) followed by salaried individuals (government or private jobs i.e. those working in the private sector) and 44.3% (n=246) were educated upto secondary level. Majority of the respondents (243, 71.9%) were smokers. **Table 2** compares the socio-demographic characteristics between those contacted versus those who could not be contacted by telephone. Significant difference in educational status was found between the groups (p =0.008).

As part of the qualitative component, a total of 26 IDIs were conducted. The socio-demographic details of the participants are given in **Table 3**. Majority of them were males (22, 84.6%), belonging to the age group 45-59 years (12, 46.2%) and were daily wage laborers (15, 57.7%). The results of the thematic analysis were categorised as: i) barriers, and ii) enablers of tobacco quitting which were further divided into three types: a) intrinsic, b)

For peer review only - http://bmjopen.bmj.com/site/about/guidelines.xhtml

extrinsic, and c) support system. The themes emerged are presented as a thematic diagram (**Figures 2 and 3**). The details of the themes, sub-themes and verbatim quotes are presented in **Table 4**.

Among those contacted and consented by telephone, 403 (72.6%) have made at least one attempt to quit, of whom 210 (52%) successfully quit, 193 (48%) made a failed quit attempt. Among those who had quit successfully, we explored the enablers for quitting smoking which are described below.

Enablers/motivators for quitting

a. Extrinsic factors

<u>*Adverse health effects*</u>

Recognition of the harms of tobacco to personal health and that of others, especially children in the family was reported to be a motivator for change.

"*It is a bad habit, it causes many diseases, children do not like the habit. It is evident that our smoking habit affects others, it affects our health also. It can affect our health, cause cough, cold and cancer*" *(65, Male)*

<u>*Responsible parents*</u>

Some men spoke of their concerns about the harms from second hand smoke (SHS) and wanted to protect their children and family.

b. Intrinsic factors

<u>*Harm to social image*</u>

Upholding social image was considered as one of the key component of enablers which helped in successful quit attempt.

"When we smoke around women in a bus stop, they frown. They cover their face with a handkerchief while I smoke. I feel bad. How much ever we act decent, the respect for people who smoke is always less. People don't respect those who smoke" (56, Male)

<u>*Benefits of quitting*</u>

Respondents found many advantages in quitting tobacco use such as being approved by their family, feeling contented from people's approval, financial benefits, and improved health.

An old 65-year old male opined, "*I was not able to eat much while using tobacco. Now I don't have any such feeling. Since I have quit, I am able to eat good amount of food. I don't have teeth stains and mouth ulcerations*"

For peer review only

Majority of the successful quitters reported that they had quit tobacco usage by their own will and determination (110, 50.7%), followed by advice from family (32, 14.7%) and advice from doctors (26, 12.0%). The least sought methods of cessation were counseling (03, 1.4%) and substitution (03, 1.4%) (**Table 5**).

The qualitative interviews also revealed that support from family and advice by doctors were the enablers for quitting smoking besides the extrinsic and intrinsic personal motivators for quitting detailed above.

c. *Support system*

Support from family

Support from children and spouse has been one of the positive reinforces which enabled successful quit attempts.

A 65-year old male said, "My wife, son and friend were against this habit. So I decided to quit. Purely my decision and my wife's support"

Support from a past quitter

Support from successful quitters has helped the respondents quit tobacco use.

"*My close friend had quit and he was supportive. He said it was good that I quit tobacco.*" (39, Male)

Health advice by doctor

Advice by doctors also prompted many to quit the habit, especially those who already have adverse physical effects of tobacco.

"*The doctor said if I continue smoking, I might die early. He advised me to quit and he said that all my internal parts have been affected to some extent. He also said that if I continue, I might get TB and other diseases. After that I felt that I should definitely quit*" *(56, Male)*

Use of substitutes

Respondents used substitutes such as chocolate, bubble gum, tulsi (*basil*) leaves to overcome craving and sustain the quit attempt.

Among current tobacco users, 115 (34.1%) did not make any quit attempt and 193 (57.3%) made a failed quit attempt. (**Figure 1**)

Those who made a failed attempt or did not make any quit attempt were interviewed in-depth to explore the extrinsic, intrinsic and other barriers in quitting smoking which are narrated below.

For peer review only

Barriers to quitting

a. Intrinsic factors

<u>*Tobacco dependence*</u>

Some current smokers talked about the ways in which smoking helped them 'cope' with adverse situations in life, such as giving comfort and relaxation at times of difficulties and thoughts to help manage personal tensions, work life problems and health issues. Consumption of tobacco allegedly helped the respondents to alleviate their pain or stress and improve digestion.

"*Some or the other tension keeps happening. Some problem keeps occurring. At that time, when you smoke it is relaxing, feels good. Smoking one cigarette reduces anger*" *(46, Male)*

<u>*No perceived health effects*</u>

Some respondents were unaware of the consequences of long-term usage of tobacco while using it spontaneously without any specific intention.

"Health will be affected. We will become weak and have heavy breathing. But I do not do deep inhaling. I smoke very lightly and throw it away. So I think I don't have much effects" (67, Male)

<u>*Craving: withdrawal symptoms*</u>

Respondents have reported strong urge to smoke and withdrawal symptoms such as nausea, vomiting, tingling sensation in mouth, headache and craving during the evenings after quitting.

b. Extrinsic factors

<u>*Availability of tobacco products*</u>

Many respondents opined that widespread availability of tobacco products makes it difficult to withhold them from usage.

"We are using because they are selling it. If they do not sell we won't use it" *(36, Male)*

<u>*Social/peer influence*</u>

Some participants expressed that the offering of cigarettes from friends and relatives was the main reason for their failure to quit.

"*Even if we stay at home wanting to stay away, when other people use, we get the craving. When others use and when they say smoke once nothing will happen, we get the urge*" *(46, male)*

c. Support system

<u>*Lack of professional help*</u>

For peer review only

Some respondents have cited lack of professional help in terms of counselling or advice as a barrier to quit as they are not confident enough to do it on their own.

A 45-year old male respondent said, "*I am unable to do it on my own. I think counselling or any sort of support would help. If possible, you can try to shut down the tobacco companies*" *(45, male)*

The quantitative survey also echoed this which said that counselling was the least sought method for cessation.

Significant association between current tobacco use and using smoking form of tobacco products (aRR=1.2, 95% CI: 1.1-1.4) and with exposure to smoke at home, which is a proxy indicator for smoking policy at home (aRR=1.2, 95% CI: 1.1-1.3) was noted (**Table 6**).

DISCUSSION

This is the first such study that we are aware of, to attempt a follow up of participants of a survey done three years before by telephone calls to understand their current tobacco use status and whether they have made any quit attempt. Only one-fourth of the respondents could be contacted by telephone. This mixed methods assessment among tobacco users of TNTS cohort found that of those contacted and consented for telephone interview, one-third of them have successfully quit tobacco in the last three years and currently are non-tobacco users. Nearly three-quarters have made any quit attempt, of whom half of them could sustain the quit attempt. The qualitative part of the study identified the reasons for failure to quit and the enablers for quitting. The key findings of the study are discussed below.

Unsurprisingly, the study reported poor response rate to a telephone survey. Only one out of four respondents could be contacted. Although telephone surveys have been used widely in public health research and market research, there are concerns regarding poor response rate both due to failure to contact and refusal to participate once contacted. A major reason for poor response rate in this study could be the fact that the contact details of the study participants were collected nearly 3 years ago when the TNTS was conducted. It is highly likely that participants would have changed their numbers which is quite common these days due to cut-throat competition in the telecom market and attractive offers by different network providers. Calls could not be made in a substantial proportion of cases, despite having a telephone number probably due to network issues, improper recording of phone number, tendency of people to switch between networks or possess more than one mobile number etc. Telephone number was not recorded in one-fourth of the respondents, meaning they either did not have any contact

number/mobile phone or did not want to share the number or the number was not recorded. These considerations should be weighed in before planning any telephone survey. Moreover, different populations might have different challenges with respect to the use of telephone/mobile phone-based surveys, which needs to understood before planning such surveys. Although telephone surveys yield poor response rates compared to household surveys which have response rates >90%, logistically telephone surveys are preferred. (21)(22)(23)

A study by Boland et al. found poor response rate as low as 17.7% in telephone surveys similar to the present study.(24) In a community based telephone survey in the USA, response rate was 37%.(25) Another study in India in 2006 using telephone survey as a method of data collection yielded a high response rate of 94%. This was probably because it was a landline telephone-based survey and during those times landline numbers did not change frequently. The study was also done in a limited geographical area in urban location covering 50 households.(26) Based on the study experience and existing literature, we suggest additional strategies such as multi-modal data collection approaches instead of using single method, incentivisation and careful interviewer selection to improve response rate. In this study, the interviewer was a trained staff and part of a call centre of a project routinely involved in making telephone calls to project participants, native of Tamil Nadu (study area) and fluent in the local language (*Tamil)*. However, nearly one-fourth of those who were contacted did not give consent for the interview, which requires additional intervention to improve participation. One such intervention was tried in Australia which concluded that mailing a postcard prior to the first telephone contact increases participation rate.(27)

One-third of the tobacco users have quit tobacco in the last three years and the remaining continue to use tobacco. This is an encouraging finding considering the poor quit rates of 5-10% across several studies. (7,8,28) However, this was self-reported and there was no objective way of assessing this response. A systematic review has shown trends of underestimation when smoking prevalence is based on self-report compared to cotinine-assessed smoking status.(29)

Nicotine addiction has been established the biggest cause of failure in smoking cessation. Tobacco dependence expressed in terms of craving for tobacco products, withdrawal symptoms, psychological dependence and habit forming emerged as the most important barriers to quitting in this study. These factors have specific management implications stressing the need for offering evidence-based tobacco cessation support including medications in line

with the MPOWER strategy. The use of smoking cessation aids in our setting has been low similar to the findings of the present study. A national survey in India revealed that nearly 90% of former smokers quit without any professional aid.(30) Participants are reluctant to receive professional help and prefer to 'quit' by themselves. Few of the respondents also reported that quitting was difficult without support and were unaware of the availability of cessation aids. Evidence based tobacco cessation methods should be available and accessible to all through a primary care delivery model. People should be made aware of these services and their role in quitting tobacco and sustaining it.

Peer influence was a major barrier to quitting tobacco as reported in other studies as well.(31–33) Offering cigarettes/tobacco to one another is perceived as a sign of friendship and this culture serves as an impediment to smoking cessation. People need to be taught methods of rejecting the offer and that declining an offer of a cigarette/tobacco is not seen to be rude.

Most of the respondents reported symptoms of tobacco withdrawal during the initial phase of quitting. At the same time, unanticipated benefits such as a feeling of wellbeing both physically and psychologically, personal satisfaction, improved social relationships, encouragement from the family were also reported, and these benefits were 'self-reinforcing' in helping them to maintain their quit status. Thus, besides the health benefits, the collateral social, economic and psychological gains should also be conveyed to those who are interested in quitting tobacco as part of the counselling package.

The study found that tobacco users with a smoke free policy at home were more likely to quit tobacco. This implies that smoke-free homes influence norms within the family around tobacco use. This inference could also be extended to other public places, thereby generating additional evidence for stricter implementation of smoke-free legislations in all public places.

The study investigators who conducted the IDIs are experienced qualitative researchers with strong interpersonal skills, which is essential in the context of telephone interview to establish rapport quickly and conduct interviews in a conversational manner. These skills helped the interviewer to work through tense and awkward moments that arose during the telephone interaction. Preparation of interviews was also done through mock trainings to handle any situation. The interviewers who work in a cancer care centre were not related to the participants nor were they involved in provision of their care directly or indirectly.

As far as we are aware, this is the first such attempt to reach out to tobacco users identified in the TNTS 2015-16 after 3 years by a telephone survey. This novel method of survey gave useful insights into the utility of telephone surveys in the Indian context and also provided understanding related to quit attempts and successful quit rates in a large cohort of tobacco users.

The study had two key limitations. The major limitation was the poor response rate of the telephone survey opted due to resource limitation which might have introduced responder bias. However, the baseline characteristics of those who were contacted versus those who could not be contacted by telephone were similar except educational status, suggesting that the results could be generalised to the entire cohort. Secondly, there was no objective means of verifying the responses received by telephone survey. However, we feel that the social desirability bias is likely to be less in a telephone conversation due to lack of face-to-face interaction.

Conclusion

Nearly two-thirds of the tobacco users have continued using it in the last 3 years. Lack of professional help and tobacco dependence were the major barriers to quitting which warrant decentralised evidence-based cessation interventions. There is evidence for the role of peer-led interventions involving family, peers and other tobacco users in quitting which could be incorporated into cessation interventions.

Recommendations

Future research can consider on-field follow-up of tobacco users, as it could yield higher response rates than telephone follow-up. Research to increase response rates in a telephone survey can also be done. Considering the number of tobacco users who have quit or expressed their willingness to quit by their own self and determination, it is high time to develop interventions involving support system including family, friends and healthcare professionals as these were reported to be major catalysts facilitating quitting of tobacco.

Acknowledgements:

This research was conducted through the Structured Operational Research and Training Initiative (SORT IT), a global partnership led by the Special Programme for Research and Training in Tropical Diseases at the World Health Organization (WHO/TDR). The model is based on a course developed jointly by the International Union Against Tuberculosis and Lung Disease (The Union) and Medécins sans Frontières (MSF/Doctors Without Borders). The

specific SORT IT programme which resulted in this publication was jointly developed and implemented by: The Union South-East Asia Office, New Delhi, India; the Centre for Operational Research, The Union, Paris, France; Medécins sans Frontières (MSF/Doctors Without Borders), India; Department of Preventive and Social Medicine, Jawaharlal Institute of Postgraduate Medical Education and Research, Puducherry, India; Department of Community Medicine, All India Institute of Medical Sciences, Nagpur, India; Department of Community Medicine, ESIC Medical College and PGIMSR, Bengaluru, India; Department of Community Medicine, Sri Manakula Vinayagar Medical College and Hospital, Puducherry, India; Karuna Trust, Bangalore, India; Public Health Foundation of India, Gurgaon, India; The INCLEN Trust International, New Delhi, India; Indian Council of Medical Research (ICMR), Department of Health Research, Ministry of Health and Family Welfare, New Delhi, India; Department of Community Medicine, Sri DevrajUrs Medical College, Kolar, India; and Department of Community Medicine, Yenepoya Medical College, Mangalore, India. We would like to thank the recruiters and the telephone interviewers for the successful conduction of the study.

Funding:

The training program, within which this paper was developed, was funded by the Department for International Development (DFID), UK. The funders had no role in study design, data collection and analysis, decision to publish, or preparation of the manuscript.

Competing interests: None declared.

Author contributions:

Conception and design, Protocol development: SV, EGSV, JPT, NK, TA, RS

Data Collection: SV, RS, DP

Data Analysis: SV, JPT, SR, RS

Drafting the paper: SV, JPT, RS, TA, MM, AN

Critical review and final approval: SV, EGSV, JPT, AK, EH, TA, NK, AN

For peer review only

References

1. WHO Report on The Global Tobacco Epidemic 2019. World Health Organisation. Geneva; 2019.
2. Drope J, Schluger NW, editors. The Tobacco Atlas. Sixth Edit. American Cancer Society; 2018.
3. Fong GT, Hammond D, Laux FL, Zanna MP, Cummings KM, Borland R, et al. The near-universal experience of regret among smokers in four countries: findings from the International Tobacco Control Policy Evaluation Survey. Nicotine Tob Res. 2004 Dec;6 Suppl 3:S341-51.
4. Benowitz NL. Nicotine addiction. N Engl J Med. 2010 Jun 17;362(24):2295–303.
5. Vangeli E, Stapleton J, Smit ES, Borland R, West R. Predictors of attempts to stop smoking and their success in adult general population samples: a systematic review. Addiction. 2011 Dec;106(12):2110–21.
6. U.S. Department of Health and Human Services. How Tobacco Smoke Causes Disease: The Biology and Behavioural Basis for Smoking - Attributable Disease. Rockville, MD; 2010.
7. Hughes JR, Keely J, Naud S. Shape of the relapse curve and long-term abstinence among untreated smokers. Addiction. 2004 Jan;99(1):29–38.
8. Tobacco Use and Dependence: A 2011 Update of Treatments [Internet]. [cited 2018 Nov 16]. Available from: https://www.medscape.org/viewarticle/757167
9. Ministry of Health & Family Welfare, GOI WHO and T. GATS-2 Global Adult Tobacco Survey Fact Sheet India 2016-17.
10. Dandona L, Dandona R, Anil Kumar G, Shukla DK, Paul VK, Balakrishnan K, et al. Nations within a nation: variations in epidemiological transition across the states of India, 1990 -2016 in the Global Burden of Disease Study. Lancet. 2017;390:2437–60.
11. Resource Centre for Tobacco Control, Cancer Institute(WIA). Tamil Nadu Tobacco Survey(TNTS) 2015-16. Chennai, India; 2016.
12. Creswell J, Plano Clark V. Designing and Conducting Mixed Methods Research. 2nd ed. Thousand Oaks, CA: Sage Publications Ltd; 2011.
13. Census. Primary Census Abstracts, Registrar General of India, Ministry of Home Affairs, Government of India. 2011.
14. Indian States by Economic Freedom - StatisticsTimes.com [Internet]. [cited 2018 Nov 16]. Available from: http://statisticstimes.com/economy/indian-states-economic-freedom.php
15. Shewade HD, Vidhubala E, Subramani DP, Lal P, Bhatt N, Sundaramoorthi C, et al. Open access tools for quality-assured and efficient data entry in a large, state-wide tobacco survey in India. Glob Health Action. 2017;
16. E. V, S. DP, C. S, E. HR, Shewade HD, Lal P. An efficient method to conduct large population survey in a low resource setting: Tamil Nadu tobacco survey. Int J Community Med Public Heal. 2017;4(12):4495.
17. Saunders B, Sim J, Kingstone T, Baker S, Waterfield J, Bartlam B, et al. Saturation in qualitative research: exploring its conceptualization and operationalization. Qual Quant. 2018;52(4):1893–907.
18. Braun V, Clarke V, Braun V, Clarke V. Applied Qualitative Research in Psychology. Appl Qual Res Psychol. 2017;0887(2006).
19. Lincoln YS, Guba EG. Naturalistic inquiry. Sage Publications; 1985. 416 p.
20. Tong A, Sainsbury P, Craig J. Consolidated criteria for reporting qualitative research

(COREQ): a 32-item checklist for interviews and focus groups. Int J Qual Heal care. 2007 Dec 16;19(6):349–57.
21. Tata Institute of Social Sciences (TISS) and Ministry of Health and Family Welfare G of I. Global Adult Tobacco Survey GATS 2 India 2016-17.
22. National Family Health Survey [Internet]. [cited 2020 May 26]. Available from: http://rchiips.org/nfhs/
23. District Level Household & Facility Survey .... [Internet]. [cited 2020 May 26]. Available from: http://rchiips.org/
24. Boland UM, Sweeney M, Scallan E, Harrington M, Staines A. Emerging advantages and drawbacks of telephone surveying in public health research in Ireland and the. 2006;
25. Krueger AB, Stone AA. Assessment of pain: a community-based diary survey in the USA. Lancet. 2008;371(9623):1519–25.
26. Thulasingam M, Cheriyath PK. Telephone Survey as a Method of Data Collection in South India. 2008;33(4):268–70.
27. Iredell H, Shaw T, Howat P, James R, Granich J. Introductory postcards: Do they increase response rate in a telephone survey of older persons? Health Educ Res. 2004;19(2):159–64.
28. Carlson S, Widome R, Fabian L, Luo X, Forster J. Barriers to Quitting Smoking Among Young Adults: The Role of Socioeconomic Status. Am J Heal Promot. 2018 Feb 7;32(2):294–300.
29. Gorber SC, Schofield-Hurwitz S, Hardt J, Levasseur G, Tremblay M. The accuracy of self-reported smoking: A systematic review of the relationship between self-reported and cotinine-assessed smoking status. Nicotine Tob Res. 2009;11(1):12–24.
30. Srivastava S, Malhotra S, Harries AD, Lal P, Arora M. Correlates of tobacco quit attempts and cessation in the adult population of India: Secondary analysis of the Global Adult Tobacco Survey, 2009-10. Vol. 13, BMC Public Health. 2013.
31. Binnal A, Rajesh GR, Ahmed J, Denny C, Nayak SU. Insights into smoking and its cessation among current smokers in india. Asian Pacific J Cancer Prev. 2013;14(5):2811–8.
32. Berkelmans A, Burton D, Page K, Worrall-Carter L. Registered Nurses' smoking behaviours and their attitudes to personal cessation. J Adv Nurs. 2011 Jul;67(7):1580–90.
33. Ransing RS, Patil DB, Desai MB, Modak A. Outcome of tobacco cessation in workplace and clinic settings: A comparative study. J Int Soc Prev Community Dent. 2016 Sep 1;6(5):487–92.

For peer review only

Table 1: Socio-demographic and characteristics of tobacco use among previously identified tobacco users in eleven selected districts during Tamil Nadu Tobacco Survey (TNTS) (2015-16) who completed the follow up survey' 2019 (N=555)

**Characteristics**	**N**	**(%)**
**Current Tobacco Use**		
Yes	338	(60.9)
No	217	(39.1)
**Type of tobacco use**		
Smoking	243	(71.9)
Smokeless	87	(25.7)
Both	8	(2.4)
**Type of tobacco smoke (n =)**		
Cigarette	151	(27.2)
Bidi	121	(21.8)
Cigar	01	(0.2)
**Type of tobacco smokeless (n =)**		
Tobacco chewing alone	08	(1.4)
Tobacco + Pan masala	68	(12.3)
Snuff	06	(4.5)
Others	35	(6.3)

For peer review only

Table 2 Comparison of socio-demographic characteristics among those contacted versus those who could not be contacted by telephone (n=2909)

Characteristics	Contacted by telephone N=555 n(%)	Could not contact by telephone N=2354 n (%)	p-value
**Age**			0.1
18-24	11 (2.0)	61 (2.6)	
25-44	250 (45.0)	1038 (44.1)	
45-64	247 (44.5)	1020 (43.3)	
≥65	47 (8.5)	235 (10.0)	
**Gender**			0.06
Male	511 (91.8)	2092 (90.6)	
Female	44 (8.2)	260 (9.4)	
**Occupation**			0.12
Unemployed: unable to work	11 (2.0)	71 (3.0)	
Unemployed: able to work	12 (2.2)	47 (2.0)	

Homemaker	25 (4.5)	151 (6.4)	
Daily wage	338 (60.9)	1349 (57.3)	
Self-employed	82 (14.8)	296 (12.6)	
Private/Govt. Job	63 (11.4)	299 (12.7)	
Missing	24 (4.3)	141 (6.0)	
**Education**			0.008
No formal school	17 (3.1)	106 (4.5)	
Primary	105 (18.9)	386 (16.4)	
Secondary	246 (44.3)	929 (39.5)	
Higher secondary and above	86 (15.5)	390 (16.6)	
Missing	101 (18.2)	543 (23.0)	
**Intention to quit***			0.1
Yes	338 (60.9)	1522 (64.5)	
No	148 (26.7)	528 (22.6)	
Missing	69 (12.4)	304 (12.9)	
**Exposure to smoke at home***			0.09
Yes	362 (65.2)	1452 (61.7)	
No	185 (33.3)	857 (36.4)	
Missing	8 (1.5)	45 (1.9)	

*from previous TNTS

Table 5 Method of cessation support sought (last attempt) to quit tobacco among those who are current non-smokers (n=210)

**Cessation method**	**N**	**(%)**

For peer review only

Counselling	03	(1.4)
NRT	05	(2.3)
Other medications	16	(7.4)
Substitution	03	(1.4)
Self (No support)	183	(87.1)
Total	210	(100)

For peer review only

Table 6: Association of socio-demographic and tobacco use related characteristics with current tobacco user status after the TNTS survey among previously identified tobacco users in 11 selected districts who completed the follow up survey 2019

Characteristics	Total N	Current tobacco user n (%)*	Non tobacco user n (%)	Unadjusted Relative Risk (95% CI)	p-value	Adjusted Relative Risk (95% CI)
**Age**						
18-24	11	5 (45.5)	6 (55.5)	0.9 (0.5-1.9)	0.8	0.8 (1.5-1.8)
25-44	245	151 (61.6)	94 (38.4)	1.3 (0.9-1.7)	0.13	1.3 (0.8-1.7)
45-64	244	158 (64.8)	86 (35.2)	1.3 (1.0-1.8)	0.05	1.3 (0.9-1.6)
≥65	47	23 (48.9)	24 (51.1)	1.0	-	1.0
**Gender**						
Male	509	313 (61.5)	196 (38.5)	1.0 (0.7-1.2)	0.8	-
Female	38	24 (63.1)	14 (36.8)	1.0	-	
**Occupation**						-
Unemployed	23	12 (52.2)	11 (47.8)	0.8 (0.5-1.3)	0.4	
Homemaker	25	14 (56.0)	11 (44.0)	0.9 (0.6-1.4)	0.6	
Daily Wage	334	212 (63.5)	122 (36.5)	1.0 (0.8-1.3)	0.8	
Self-employed	79	49 (62.0)	30 (38.0)	1.0 (0.8-1.3)	0.9	

For peer review only

Private/Govt job	62	38 (61.3)	24 (38.7)	1.0	-	
**Previous tobacco use**						
Smoking	395	251 (63.5)	144 (36.5)	1.2 (1.1-1.4)	0.04	1.2 (1.1-1.4)*
Smokeless	160	87 (54.4)	73 (45.6)	1.0	-	
**Previous intention to quit**						
Yes	144	82 (56.9)	62 (43.1)	1.0	-	1.0
No	336	214 (63.3)	122 (36.3)	0.9 (0.8-1.1)	0.09	0.9 (0.9-1.2)
**Exposure to smoke at home$^{\beta}$**						
Yes	164	113 (68.9)	51 (31.1)	1.2 (1.1-1.4)	0.008	1.2 (1.1-1.3)*
No	313	182 (58.1)	131 (41.9)	1.0	-	1.0

$^{\beta}$captured during TNTS survey *row percentage; education was removed because it had high multi-collinearity with occupation analysis has been adjusted for clustering at the district level.

For peer review only

Table 3 Socio-demographic characteristics of participants of In-depth interviews, 2019

Characteristics	Frequency	Percentage
**Gender**		
Male	22	84.6
Female	4	15.4
**Age**		
18-24	2	7.7
25-44	9	34.6
45-59	12	46.2
≥60	3	11.5
**Occupation**		
Homemaker	3	11.5
Daily wage	15	57.7
Self-employed	5	19.2
Private/Govt. Job	3	11.5
**Education**		

For peer review only

Primary	2	7.7
Secondary	16	61.5
Higher secondary and above	5	19.2
Missing	3	11.5
**Quit attempt**		
Successful attempt	10	38.4
Failed attempt	10	38.4
Did not attempt	6	23.1

For peer review only

Table 4 Themes and sub-themes of enablers and barriers of quitting tobacco and sustaining it with corresponding quotes

	Theme	Sub-theme		Quotes
Enablers	Extrinsic	Adverse health effects: Self & Others		*"It affects everything. It is a bad habit. It is harmful to health. I get cough, cold. All the internal organs are affected because of this. Quitting this is a very good deed"*
		Responsible parents		"*Doctors are saying that it affects the children immediately. All I want is children should not be affected, people at home should respect me and I should not have cough anymore. When my children said quit this, I decided to quit*"
	Intrinsic	Harm to social image		*"People around us used to frown when we are using tobacco next to them. I used to think whether it is such a horrible thing"*
		Benefits of quitting	Immediate effects	*"That is a very satisfying thing for me. I don't have any cough or cold after quitting"*
			Feel happy and satisfied	*"I am feeling good now. Because, I was addicted to a bad habit, but I have quit now. I feel that it's a good thing"*
			Perceived health benefits	*"Used to get cold, cough and would feel suffocated when smoking. Now after quitting, I am able to breathe normally. I am not getting exhausted now. I am able to feel that clearly. I am feeling happy that I quit"*
			Improved social and family relationships	*"I don't have cough. Now I can play with children. Initially I used to have a guilt that I keep coughing while playing with*

For peer review only

				children"
			Financial gains	*"When I am spending the 30 or 40 rupees from not purchasing cigarettes, for the sake of my children, I feel happy"*
	Support system	Support from family		*"Family was very supportive. They always advise not to drink and not to smoke. Wife fights, daughter fights. It's a problem for everyone"*
		Support from past quitter		*"My friend advised that it would be beneficial to quit and that someone would be motivated to quit after seeing me"*
		Health advice by a doctor		*"Doctor advised me not to use this tobacco. I checked with him because I had burning sensation in the chest. Doctor said that it might be because of the tobacco that I use and advised me to reduce it"*
		Use of substitutes		*"I used to take tobacco after tea. Now as soon as I have tea I keep something in my mouth. I get the craving when I see people using tobacco, but I take vicks tablet at that time"*
Barriers	Intrinsic	Tobacco Dependence	Coping with personal issues	*"I smoke definitely when I am tensed. I smoke two to three cigarettes at a time when I am angry. If people make me angry, I will smoke to relax myself"*
			Pain/ Stress reliever	*"I use it occasionally, when I have toothache. Otherwise I won't. Only for toothache"*

For peer review only

		Improves digestion	*"I smoke only one cigarette after food. I use it for better digestion, that's it"*
	Casual usage: No perceived health effects		*"I will have such effects only if I use tobacco every day. But I use it only when I have toothache. So I don't have any effects"*
	Habitual user		*"What to do… Since it has become a habit for so long, I am unable to quit"*
	Craving: Withdrawal symptoms		*"When I am in the middle of a conversation, at times, I have this craving suddenly and I feel like I have to go immediately. I am unable to control the urge"*
Extrinsic	Availability of tobacco products		*"We are using this because it's available in the shops. Also, when someone smokes and exhales in front us, we get the craving"*
	Social/ Peer influence		*"Even if I stay at home trying to not use tobacco, I would want to use when someone who is using tobacco comes and says, just use it once"*
Support system			*"If I am to quit, I will have to do it on my own will. Counselling or any sort of advice from others will not help in this case. Even when my family advices me, I move away from that place. I can quit, only if I make that decision on my own"*

For peer review only

Figure 1a Standard Operating Procedure for making telephone calls

The telephonic calls were placed by a trained project staff

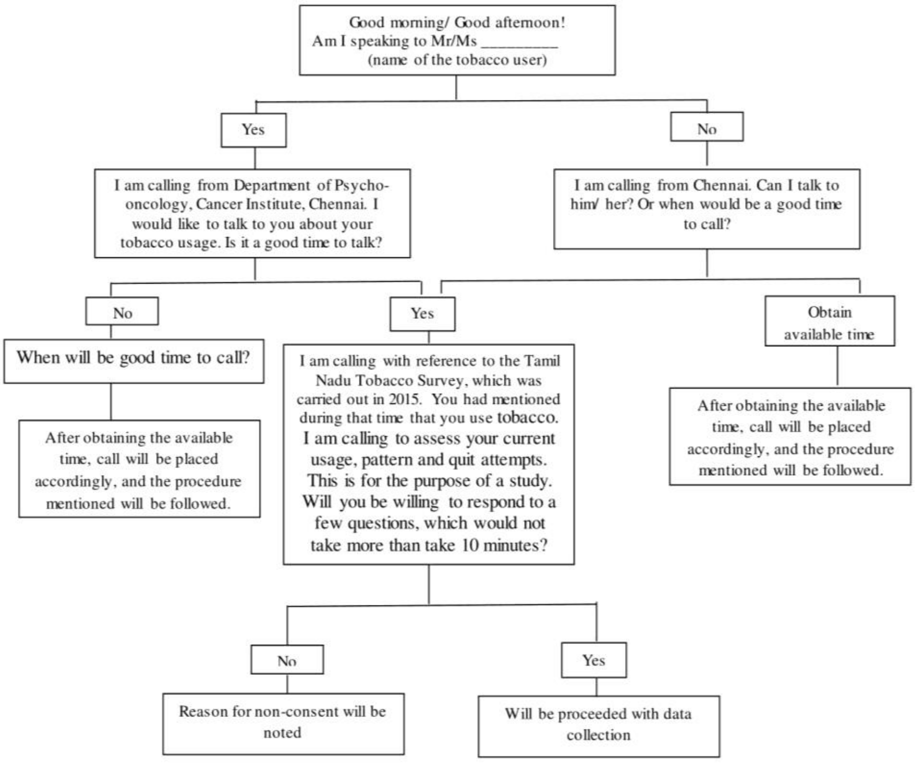

Figure 1b: Flow diagram depicting the status of current tobacco use and the pattern of quit attempts among tobacco users in six selected districts of Tamil Nadu previously identified in the Tamil Nadu Tobacco Survey (TNTS) (2015-16)

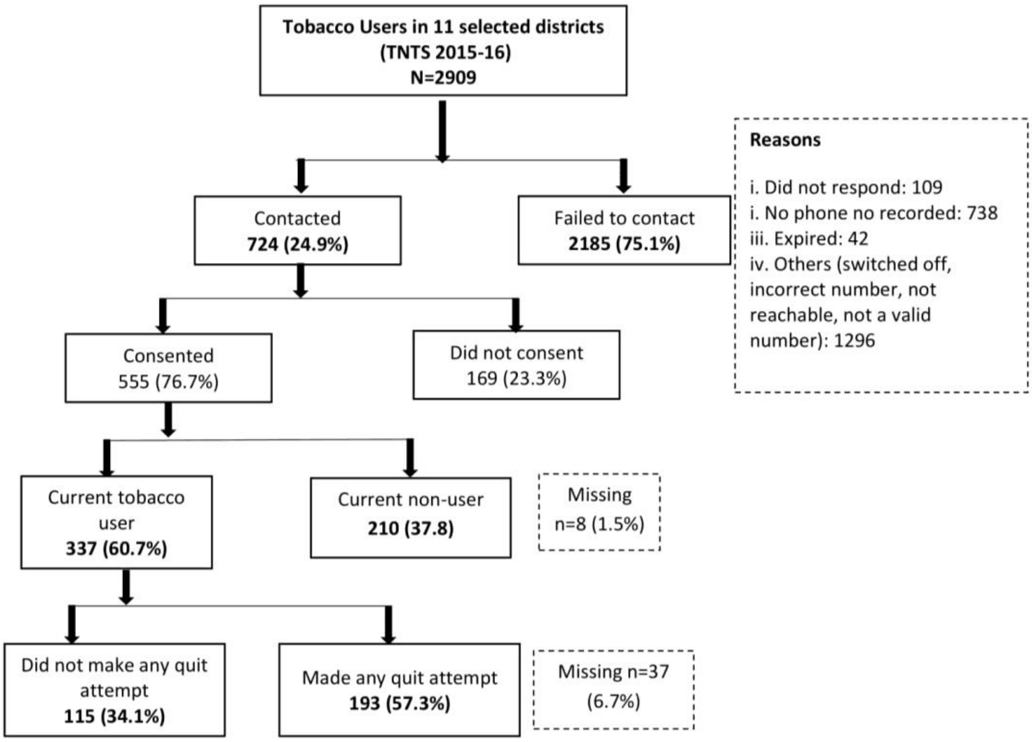

Figure 2 Enablersof quitting tobacco and sustaining it among the tobacco users in three selected districts of Tamil Nadu, 2019

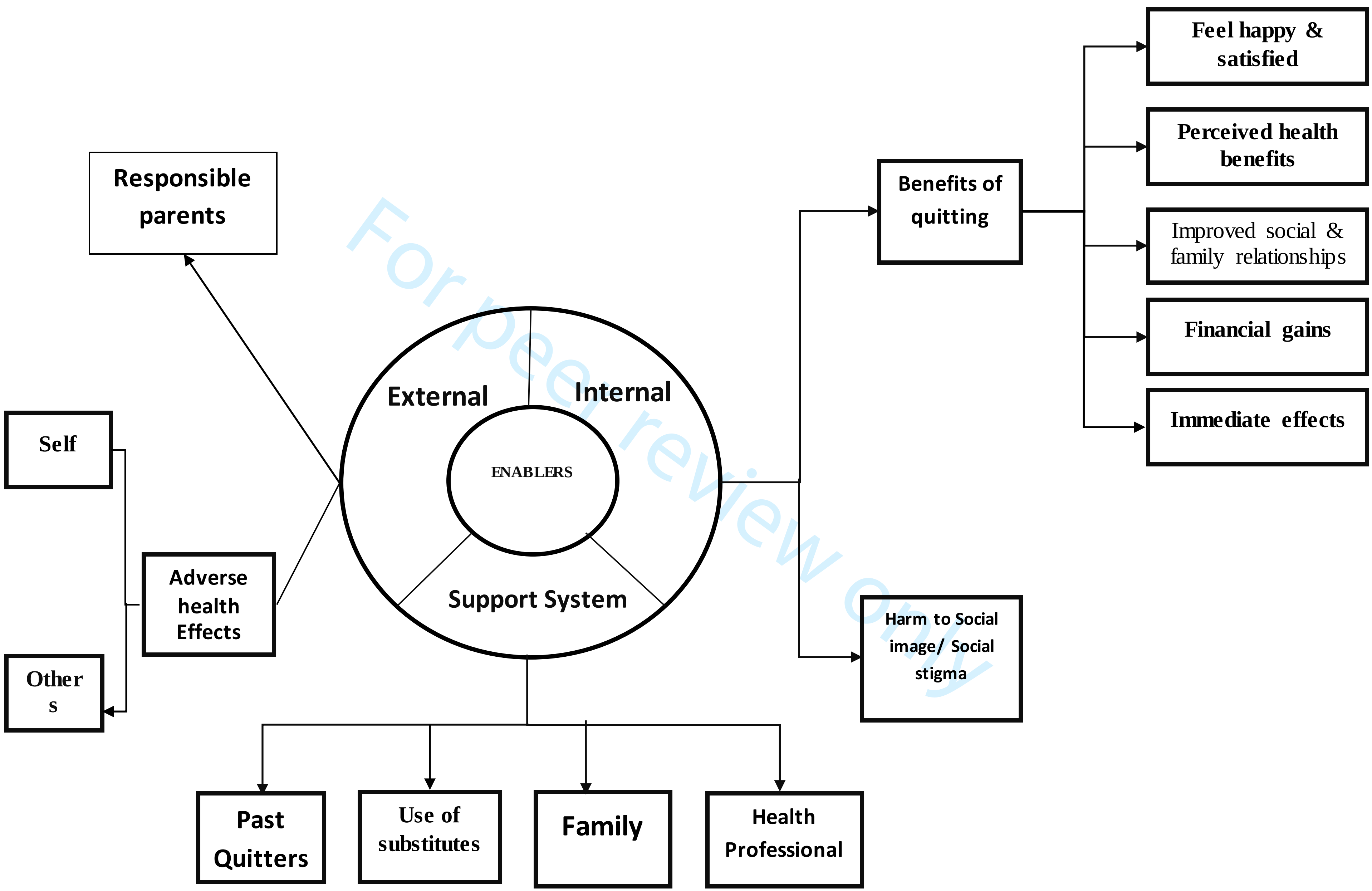

For peer review only

Figure 3 Barriers of quitting tobacco and sustaining it among the tobacco users in three selected districts of Tamil Nadu, 2019

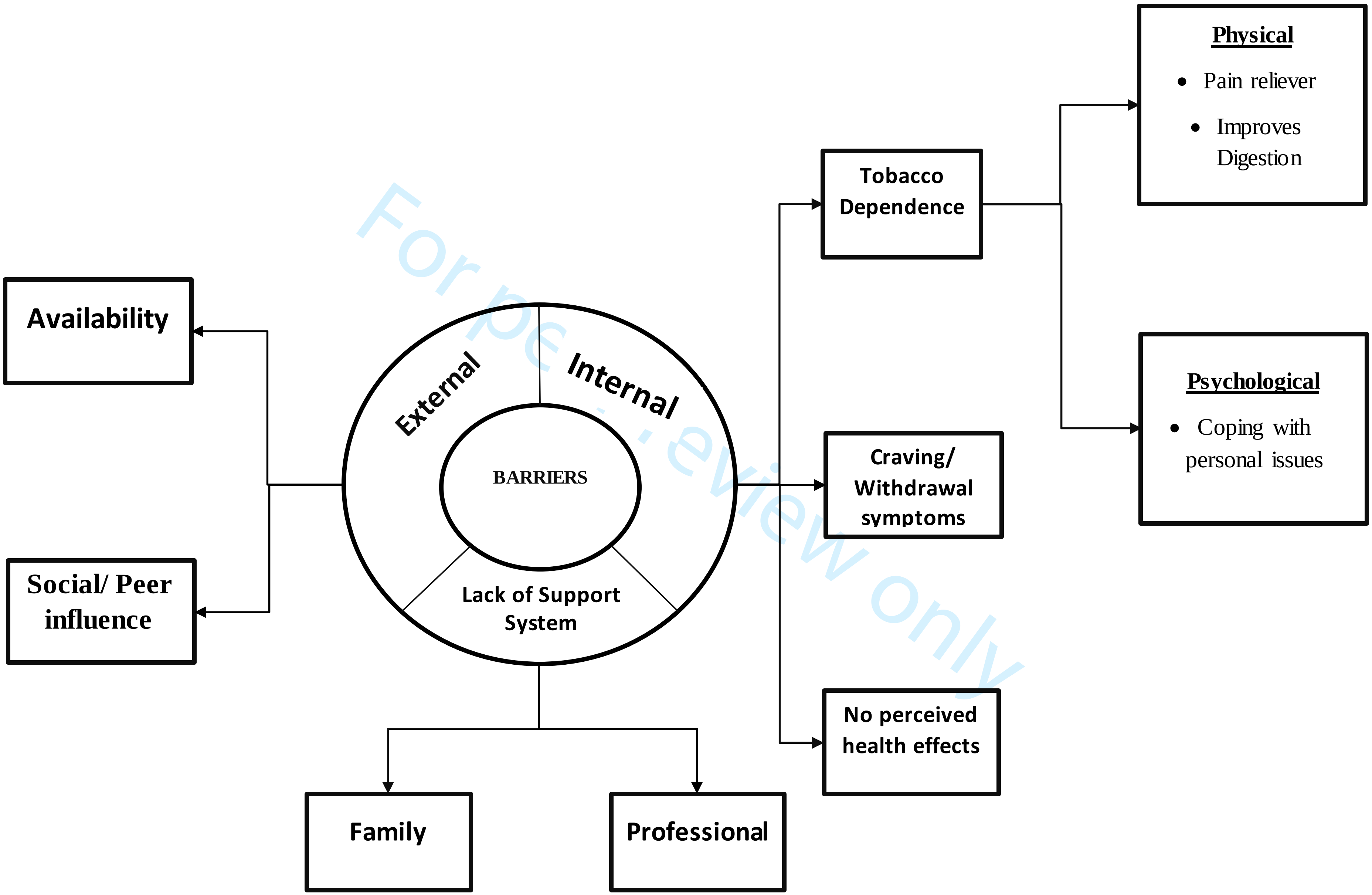

For peer review only - http://bmjopen.bmj.com/site/about/guidelines.xhtml

STROBE Statement—Checklist of items that should be included in reports of ***cohort studies***

	Item No	Recommendation	Page No
**Title and abstract**	1	(*a*) Indicate the study's design with a commonly used term in the title or the abstract	1
		(*b*) Provide in the abstract an informative and balanced summary of what was done and what was found	3
**Introduction**			
Background/rationale	2	Explain the scientific background and rationale for the investigation being reported	4
Objectives	3	State specific objectives, including any prespecified hypotheses	5
**Methods**			
Study design	4	Present key elements of study design early in the paper	6
Setting	5	Describe the setting, locations, and relevant dates, including periods of recruitment, exposure, follow-up, and data collection	6
Participants	6	(*a*) Give the eligibility criteria, and the sources and methods of selection of participants. Describe methods of follow-up	6
		(*b*) For matched studies, give matching criteria and number of exposed and unexposed	
Variables	7	Clearly define all outcomes, exposures, predictors, potential confounders, and effect modifiers. Give diagnostic criteria, if applicable	7
Data sources/ measurement	8*	For each variable of interest, give sources of data and details of methods of assessment (measurement). Describe comparability of assessment methods if there is more than one group	7
Bias	9	Describe any efforts to address potential sources of bias	
Study size	10	Explain how the study size was arrived at	7
Quantitative variables	11	Explain how quantitative variables were handled in the analyses. If applicable, describe which groupings were chosen and why	7
Statistical methods	12	(*a*) Describe all statistical methods, including those used to control for confounding	9
		(*b*) Describe any methods used to examine subgroups and interactions	
		(*c*) Explain how missing data were addressed	
		(*d*) If applicable, explain how loss to follow-up was addressed	
		(*e*) Describe any sensitivity analyses	
**Results**			
Participants	13*	(a) Report numbers of individuals at each stage of study—eg numbers potentially eligible, examined for eligibility, confirmed eligible, included in the study, completing follow-up, and analysed	10
		(b) Give reasons for non-participation at each stage	10
		(c) Consider use of a flow diagram	10
Descriptive data	14*	(a) Give characteristics of study participants (eg demographic, clinical, social) and information on exposures and potential confounders	10
		(b) Indicate number of participants with missing data for each variable of interest	10
		(c) Summarise follow-up time (eg, average and total amount)	10
Outcome data	15*	Report numbers of outcome events or summary measures over time	10

For peer review only

For peer review only - http://bmjopen.bmj.com/site/about/guidelines.xhtml

Main results	16	(*a*) Give unadjusted estimates and, if applicable, confounder-adjusted estimates and their precision (eg, 95% confidence interval). Make clear which confounders were adjusted for and why they were included<br>(*b*) Report category boundaries when continuous variables were categorized<br>(*c*) If relevant, consider translating estimates of relative risk into absolute risk for a meaningful time period	11
Other analyses	17	Report other analyses done—eg analyses of subgroups and interactions, and sensitivity analyses	
**Discussion**			
Key results	18	Summarise key results with reference to study objectives	16
Limitations	19	Discuss limitations of the study, taking into account sources of potential bias or imprecision. Discuss both direction and magnitude of any potential bias	16
Interpretation	20	Give a cautious overall interpretation of results considering objectives, limitations, multiplicity of analyses, results from similar studies, and other relevant evidence	16
Generalisability	21	Discuss the generalisability (external validity) of the study results	16
**Other information**			
Funding	22	Give the source of funding and the role of the funders for the present study and, if applicable, for the original study on which the present article is based	17

For peer review only

COREQ (COnsolidated criteria for REporting Qualitative research) Checklist

A checklist of items that should be included in reports of qualitative research. You must report the page number in your manuscript where you consider each of the items listed in this checklist. If you have not included this information, either revise your manuscript accordingly before submitting or note N/A.

Topic	Item No.	Guide Questions/Description	Reported on Page No.
**Domain 1: Research team and reflexivity**			
Personal characteristics			
Interviewer/facilitator	1	Which author/s conducted the interview or focus group?	8
Credentials	2	What were the researcher's credentials? E.g. PhD, MD	M.Phil., PhD & M.Phil
Occupation	3	What was their occupation at the time of the study?	1
Gender	4	Was the researcher male or female?	
Experience and training	5	What experience or training did the researcher have?	8
Relationship with participants			
Relationship established	6	Was a relationship established prior to study commencement?	6
Participant knowledge of the interviewer	7	What did the participants know about the researcher? e.g. personal goals, reasons for doing the research	8
Interviewer characteristics	8	What characteristics were reported about the inter viewer/facilitator? e.g. Bias, assumptions, reasons and interests in the research topic	8
**Domain 2: Study design**			
Theoretical framework			
Methodological orientation and Theory	9	What methodological orientation was stated to underpin the study? e.g. grounded theory, discourse analysis, ethnography, phenomenology, content analysis	9
Participant selection			
Sampling	10	How were participants selected? e.g. purposive, convenience, consecutive, snowball	6
Method of approach	11	How were participants approached? e.g. face-to-face, telephone, mail, email	8
Sample size	12	How many participants were in the study?	8
Non-participation	13	How many people refused to participate or dropped out? Reasons?	10
Setting			
Setting of data collection	14	Where was the data collected? e.g. home, clinic, workplace	8
Presence of non-participants	15	Was anyone else present besides the participants and researchers?	8
Description of sample	16	What are the important characteristics of the sample? e.g. demographic data, date	10
Data collection			
Interview guide	17	Were questions, prompts, guides provided by the authors? Was it pilot tested?	8
Repeat interviews	18	Were repeat inter views carried out? If yes, how many?	
Audio/visual recording	19	Did the research use audio or visual recording to collect the data?	8
Field notes	20	Were field notes made during and/or after the inter view or focus group?	8
Duration	21	What was the duration of the inter views or focus group?	8

For peer review only

Data saturation	22	Was data saturation discussed?	8
Transcripts returned	23	Were transcripts returned to participants for comment and/or	8

Topic	Item No.	Guide Questions/Description	Reported on Page No.
		correction?	
**Domain 3: analysis and findings**			
Data analysis			
Number of data coders	24	How many data coders coded the data?	9
Description of the coding tree	25	Did authors provide a description of the coding tree?	11
Derivation of themes	26	Were themes identified in advance or derived from the data?	9
Software	27	What software, if applicable, was used to manage the data?	9
Participant checking	28	Did participants provide feedback on the findings?	8
Reporting			
Quotations presented	29	Were participant quotations presented to illustrate the themes/findings? Was each quotation identified? e.g. participant number	11
Data and findings consistent	30	Was there consistency between the data presented and the findings?	15
Clarity of major themes	31	Were major themes clearly presented in the findings?	11
Clarity of minor themes	32	Is there a description of diverse cases or discussion of minor themes?	11

For peer review only

Developed from: Tong A, Sainsbury P, Craig J. Consolidated criteria for reporting qualitative research (COREQ): a 32-item checklist for interviews and focus groups. *International Journal for Quality in Health Care*. 2007. Volume 19, Number 6: pp. 349 – 357

Once you have completed this checklist, please save a copy and upload it as part of your submission. DO NOT include this checklist as part of the main manuscript document. It must be uploaded as a separate file.

For peer review only
